# High‐quality chromosome‐level genome assembly and multi‐omics analysis of rosemary (*Salvia rosmarinus*) reveals new insights into the environmental and genome adaptation

**DOI:** 10.1111/pbi.14305

**Published:** 2024-02-16

**Authors:** Yong Lai, Jinghua Ma, Xuebin Zhang, Xiaobo Xuan, Fengyun Zhu, Shen Ding, Fude Shang, Yuanyuan Chen, Bing Zhao, Chen Lan, Turgay Unver, George Huo, Ximei Li, Yihan Wang, Yufang Liu, Mengfei Lu, Xiaoping Pan, Deshuang Yang, Mingwan Li, Baohong Zhang, Dangquan Zhang

**Affiliations:** ^1^ College of Forestry Henan Agricultural University Zhengzhou Henan China; ^2^ State Key Laboratory of Crop Stress Adaptation and Improvement, Henan Joint International Laboratory for Crop Multi‐Omics Research, School of Life Sciences Henan University Kaifeng Henan China; ^3^ Key Laboratory of Water Management and Water Security for Yellow River Basin Ministry of Water Resources Zhengzhou Henan China; ^4^ School of Biological and Food Processing Engineering Huanghuai University Zhumadian Henan China; ^5^ College of Life Science Henan Agricultural University Zhengzhou Henan China; ^6^ Ficus Biotechnology Ankara Turkey; ^7^ Department of Biology East Carolina University Greenville North Carolina USA

**Keywords:** *Salvia rosmarinus*, genome assembly, oxidative stress, environmental adaptation

## Abstract

High‐quality genome of rosemary (*Salvia rosmarinus*) represents a valuable resource and tool for understanding genome evolution and environmental adaptation as well as its genetic improvement. However, the existing rosemary genome did not provide insights into the relationship between antioxidant components and environmental adaptability. In this study, by employing Nanopore sequencing and Hi‐C technologies, a total of 1.17 Gb (97.96%) genome sequences were mapped to 12 chromosomes with 46 121 protein‐coding genes and 1265 non‐coding RNA genes. Comparative genome analysis reveals that rosemary had a closely genetic relationship with *Salvia splendens* and *Salvia miltiorrhiza*, and it diverged from them approximately 33.7 million years ago (MYA), and one whole‐genome duplication occurred around 28.3 MYA in rosemary genome. Among all identified rosemary genes, 1918 gene families were expanded, 35 of which are involved in the biosynthesis of antioxidant components. These expanded gene families enhance the ability of rosemary adaptation to adverse environments. Multi‐omics (integrated transcriptome and metabolome) analysis showed the tissue‐specific distribution of antioxidant components related to environmental adaptation. During the drought, heat and salt stress treatments, 36 genes in the biosynthesis pathways of carnosic acid, rosmarinic acid and flavonoids were up‐regulated, illustrating the important role of these antioxidant components in responding to abiotic stresses by adjusting ROS homeostasis. Moreover, cooperating with the photosynthesis, substance and energy metabolism, protein and ion balance, the collaborative system maintained cell stability and improved the ability of rosemary against harsh environment. This study provides a genomic data platform for gene discovery and precision breeding in rosemary. Our results also provide new insights into the adaptive evolution of rosemary and the contribution of antioxidant components in resistance to harsh environments.

## Introduction

Plants are always accompanied by different environmental stresses, including both biotic stresses, such as pathogen and pest infection, and abiotic stresses, such as drought and salt stresses as well as nutrient deficiency, which seriously affect plant growth and development as well as crop yield and quality (Gupta *et al*., 2020; Prakash *et al*., 2023; Zhu, 2016; Zhang *et al*., 2022). To cope with these stresses, over their long evolutionary progress, plants have been developing complicated defence mechanisms, including altering structure and morphology as well as synthesizing specific metabolites and proteins. Certain halophytes form special salt sacs or glands to reduce excessive salt ions in the plant cells (Shabala *et al*., 2014; Zou *et al*., 2017); certain plants enhance their drought tolerance by regulating stomatal opening through modulating the osmotic potential of guard cells (Dong, Bai *et al*., 2018; Ren *et al*., 2018). There are also certain plants using endophytes to improve their stress resistance (Giauque *et al*., 2019). Except these, secondary metabolites also play important roles in plant adaptation to the harsh environments (Dixon, 2001; Kessler and Kalske, 2018; Pang *et al*., 2021). Many aromatic plants are rich in diverse natural antioxidant components, such as phenolic acids, terpenoids, and flavonoids, which help them cope with adverse environments (Liu, Wu *et al*., 2021; Nakabayashi *et al*., 2014). The ability of plants to adapt to complex and variable environments is not only derived from a single strategy, but is usually a comprehensive performance of multiple strategies, which is also the result of long‐term natural evolution (Baduel *et al*., 2019; Jiao and Schneeberger, 2020; Shah *et al*., 2020; Wang *et al*., 2020).

Rosemary (*Salvia rosmarinus*, synonym *Rosmarinus officinalis*) belongs to Lamiaceae family and is a perennial aromatic shrub native to the Mediterranean coastal areas of Europe and North Africa. It has been cultivated and utilized for thousands of years (Carrubba *et al*., 2020; Ribeiro‐Santos *et al*., 2015). Rosemary has relatively high tolerance to abiotic stresses and is easy to propagate from cuttings with low demand for water and fertilizer, which makes its cultivation management and harvesting easier. Given its high economic value, rosemary has been widely grown worldwide. Because of rosemary's great ecological effects, such as repelling insects, inhibiting weed growth, and maintaining water and soil, it has been widely used as an interplanting crop in economic forests such as *Camellia oleifolia* (Chen *et al*., 2016).

Rosemary originally grows on the beach and rocks in a harsh environment, which is often stressed by drought, heat, salt and other changeably unfavourable environments. To cope with these harsh environments, rosemary has evolved several strategies, including the biosynthesis of a high amount of secondary metabolites associated with antioxidant capacity (Loussouarn *et al*., 2017). The main antioxidant components in rosemary include water‐soluble components such as rosmarinic acid and its derivatives; flavonoids and lipid‐soluble components such as carnosic acid, carnophenolic and rosmarinol; and volatile components such as pinene, camphene and cineole (Pedro *et al*., 2016; Ribeiro‐Santos *et al*., 2015). The contents of these antioxidant components are important quality traits in rosemary. Additionally, the volatile components of rosemary have an obvious deterrence effect or toxic effect on insects and diseases, and effectively reduce the damage caused by encroachment (Ahmed *et al*., 2021; Gershenzon and Dudareva, 2007; Holopainen and Gershenzon, 2010; Krzyśko‐Lupicka *et al*., 2019). The high content of antioxidant components in rosemary has been well exploited. At present, rosemary has been considered to be one of the most popular sources of natural antioxidant components and the most studied Mediterranean herbs (Kaur *et al*., 2023; Sánchez‐Camargo and Herrero, 2017). The high‐activity antioxidant components in rosemary are regarded as one of the best natural antioxidant additions (Abbaszadeh *et al*., 2020; Bendif *et al*., 2017; Jessy *et al*., 2016). Rosemary leaves are widely used to extract essential oils because of its high‐content VOCs (Ribeiro‐Santos *et al*., 2015). Based on its aromatic characteristics and health benefits, rosemary has been widely used in cooking, food preservation, and, in addition, cosmetics, aromatherapy, and biomedicine (Allegra *et al*., 2020; Bajalan *et al*., 2017; Jaglanian *et al*., 2020; Ribeiro‐Santos *et al*., 2015; Sirocchi *et al*., 2017).

Because of its high content and activity of antioxidant components in rosemary (Calderón‐Oliver and Ponce‐Alquicira, 2021; Cedeño‐Pinos *et al*., 2020; Li *et al*., 2021), it will greatly increase the demand for the yield and quality of rosemary in the fields of natural antioxidant replacement, high‐quality cosmetic processing, and modern aromatic medicine. However, the existing varieties in the world could not meet this new demand. The adaptation mechanism of rosemary to complex and changeable environments is still unclear, which limits the breeding and utilization of new varieties with high‐stress resistance and high antioxidant component content. Additionally, the regulation mechanism of antioxidant component biosynthesis has not been well analysed in rosemary. A high quality of rosemary genome is helpful to solve those problems.

Initially, Nolan *et al*. (2020) employed the second‐generation sequencing technology to briefly assemble rosemary genome. Then, a high‐quality genome of rosemary was assembled by Han *et al*. (2023), mainly discussing carnosic acid biosynthesis, rather than the environmental adaptability. The environmental adaptation has a significant impact on the yield and quality of rosemary, while the molecular mechanism of rosemary's environmental adaptation is still not well revealed. Therefore, based on stress resistance identification, the line ‘Albus‐2’ with strong resistance, from the sexual progeny of the main rosemary cultivar ‘Albus’ in China, was selected. In order to reveal the environmental adaptation mechanism of rosemary and provide a genomic data platform for gene discovery and functional identification, we employed long‐read Nanopore sequencing and Hi‐C sequencing, to assemble the chromosome‐level genome of rosemary; at the same time, we employed multi‐omics analysis to interpret the inner nature of rosemary rich in antioxidant components and reveal the response mechanism to abiotic stresses. The results will gain a new insight that antioxidant components play an important role in rosemary environmental adaptability, which provides important resources for gene discovery and breeding. For antioxidant‐bearing crops, regulating the contents of antioxidant components could enhance plant resistance to achieve stable yield and high quality.

## Results

### Rosemary genome assembly and annotation

**Figure 1:**
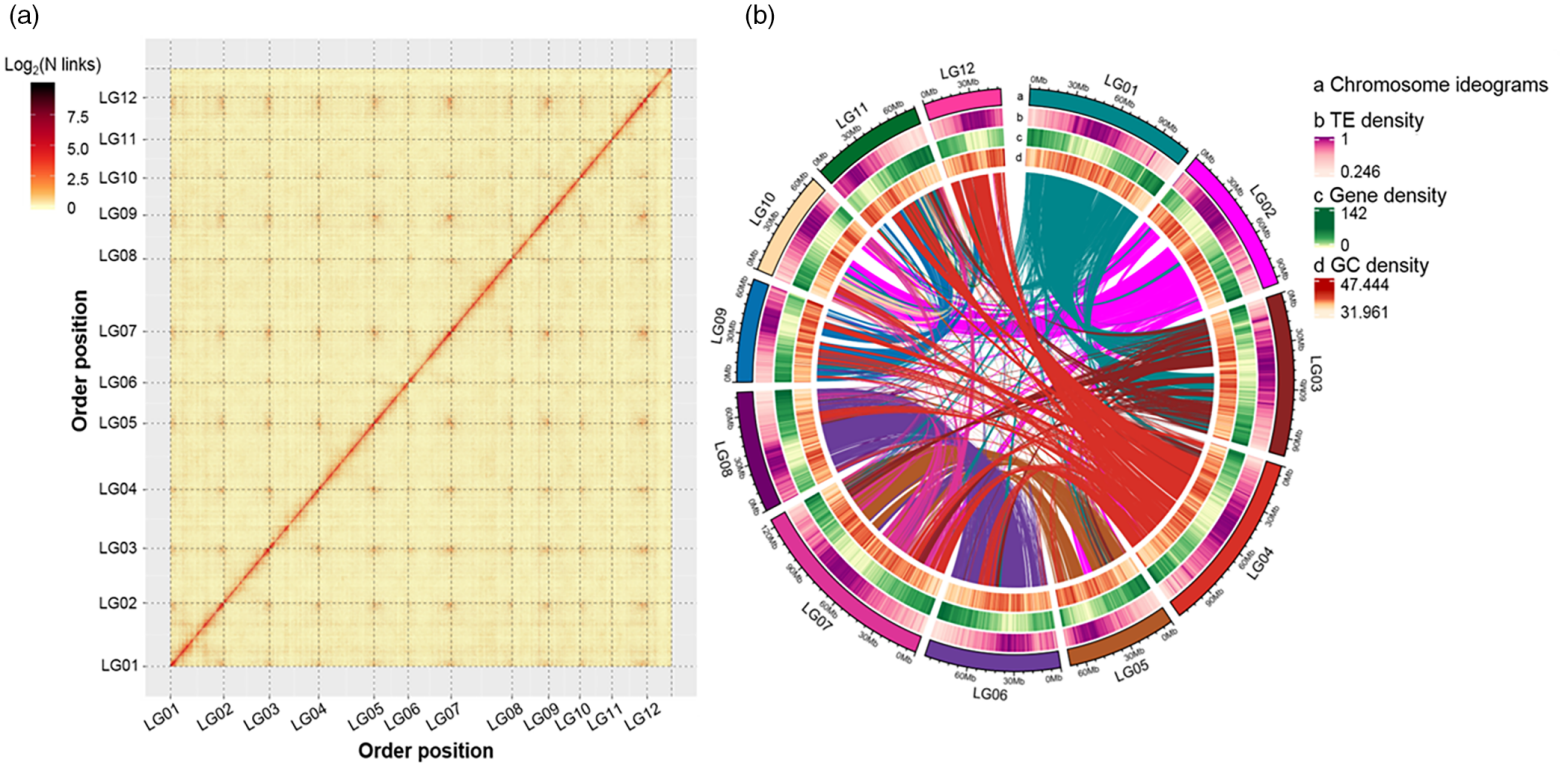
Overview of the rosemary genome assembly. (a) Hi‐C map of the rosemary genome showing a high‐quality assembled genome. (b) Overview of assembled rosemary genome.

In order to obtain more reliable genomic information of rosemary, both Nanopore and Hi‐C sequencing technologies were employed to sequence and assembly rosemary genome. A total of 135.63 Gb Nanopore sequencing data (103X sequencing depth), and 123.76 Gb clean data were obtained after filtering, with an average read length of 26.76 kb, containing a total of 4 624 981 reads. We obtained a total size of 1.20 Gb high‐quality genome, with the Contig N50 of 1.07 Mb and scaffold N50 of 87.94 Mb (Figure 1 and Table 1). After Hi‐C assembly, a total of 1.17 Gb (97.96%) of genome sequences were mapped to 12 chromosomes, of which there were 1.03 Gb sequences with defined order and orientation. Meanwhile, the Hi‐C assembly chromosome interaction heat map shows that all bins could be assigned to the corresponding 12 chromosomes (Figure 1). Among the sequences obtained by Illumina HiSeq sequencing, 99.50% of them were aligned to the assembled genome. A total of 1553 (96.22%) core genes in BUSCO were mapped to the genome (Table S1). These results indicate that a high‐quality genome of rosemary was achieved at the chromosome‐level.

**Table 1:** Summary of the rosemary genome

Assembly feature	Statistic
Genome size (Gb)	1.20
Mapped reads (%)	99.50
Number of contig N50	2194
Contig N50 (Mb)	1.07
Longest contig (Mb)	9.55
Number of scaffolds	907
Scaffold N50 (Mb)	87.94
Longest scaffold (Mb)	124.95
GC content (%)	37.92
Number of genes	46 121
Total gene length (bp)	157 545 038
Average of gene length (bp)	3415.91
Average length per exon (bp)	1298.43
Average exons per gene	5.71
Non‐coding RNAs	1265
Pseudogenes	2829

In the assembled rosemary genome, there are 924.32 Mb (77.17%) repeat sequences. Based on *de novo* prediction, unigene homologous species prediction and RNA‐seq, a total of 46 121 putative genes were achieved, and 95.10% of them were functionally annotated successfully (Table S1 and Data Set S1). A total of 1265 non‐coding RNAs were identified, including microRNAs, rRNAs and tRNAs (Table S1). In addition, 2829 pseudogenes were also identified (Table S1). Based on the conserved sequence analysis, 4598 motifs and 50 548 domains were obtained. Therefore, the high‐quality genome of rosemary was assembled and well annotated. The genome size of rosemary (cultivar Albus‐2, 1.20 Gb) is obviously larger than other *genomes* of *Salvia*, including *Salvia splendens* (0.81 Gb) (Dong, Xin *et al*., 2018), *Salvia miltiorrhiza* (0.62 Gb) (Ma *et al*., 2021), *Salvia officinalis* (0.48 Gb) (Li *et al*., 2022) and *Salvia rosmarinus* (cultivar Morcco, 1.11 Gb). The results provide a useful data platform for studying the evolution of rosemary and the functional identification of genes.

### Comparative genomic and phylogenomic analyses provide insights into the evolution

**Figure 2:**
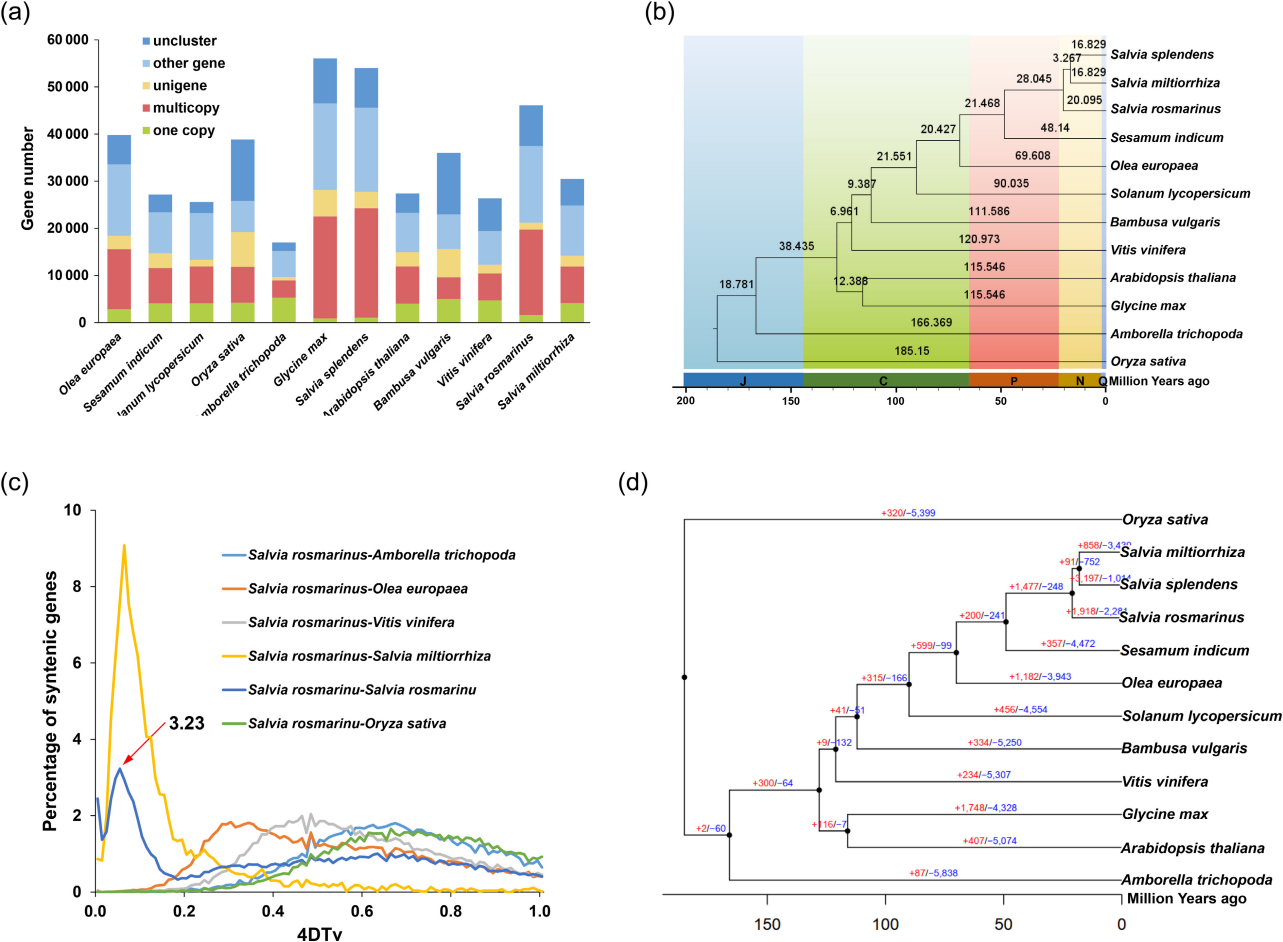
Comparative genomic analysis of rosemary genome. (a) Comparative analysis of orthologous gene families among 12 species. (b) Estimated divergence time and phylogenetic tree constructed by orthologous single‐copy genes. (c) 4DTv distance distribution. The red arrow indicates the peak value for rosemary. (d) Expansion and contraction of gene families among 12 plant genomes. Q, Quaternary; N, Neogene; P, Paleogene; C, Cretaceous; J, Jurassic.

Based on the genome assembly, we conducted comparative genomic and phylogenomic analyses to understand the evolution of rosemary. In rosemary genome, 37 464 genes were assigned to 15 968 gene families. According to gene family clustering analysis, there were 583 specific gene families containing 1459 specific genes unique to rosemary compared with other 11 species, including *Arabidopsis thaliana*, *Olyza sativa* and *Salvia splendens* (Figure 2a and Table S2). The single‐copy protein sequences of rosemary and the other 11 species were used to construct the evolutionary tree. The results showed that rosemary had a closely genetic relationship with *S. splendens* and *S. miltiorrhiza*, and it diverged from them approximately 33.7 million years ago (MYA) (Figure 2b). The fourfold synonymous third‐codon transversion (4DTv) analysis shows that one whole‐genome duplication (WGD) occurred around 28.3 MYA in rosemary genome (Figure 2c). LTR insertion time analysis show that an LTR insertion event occurred about a million years ago (Figure S1). During rosemary genome evolution, 1918 gene families were expanded (Table S2), 35 of which are involved in the biosynthesis of antioxidant components, and 2281 families contracted in the rosemary genome (Figure 2d). Gene Ontology (GO) annotation analysis shows that the majority of the expanded genes were involved in metabolic processes (Figure S2 and Data Set S2). According to functional annotation, the positive selective genes were mostly involved in biological processes such as metabolic processes and response to stimuli (Figure S3 and Data Set S3). These results indicate that metabolic processes play an important role in the environmental adaptation of rosemary.

### Expanded gene families associated with environmental adaptation in rosemary

**Figure 3:**
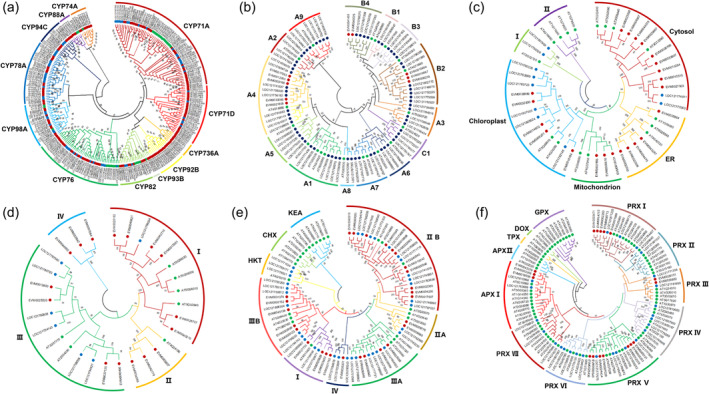
Phylogenetic tree of CYPs (a), HSF (b), HSP70 (c), HSP90 (d), potassium transporters (e) and peroxidase (f) based on the protein sequence alignments from rosemary (red solid circles), *Salvia splendens* (blue solid circles) and *Arabidopsis* (green solid circles) using the MEGA X with the neighbour‐joining methods. HSP, heat shock protein; HSF, heat stress transcription factor.

The expansion of gene families may result in gene dosage effects and enhance certain phenotypes, which plays an important role in species evolution and environmental adaptation (Stortenbeker and Bemer, 2018; Waters and Vierling, 2020; Yun *et al*., 2022). To gain more insights into rosemary's environmental adaptation, the expanded gene families were analysed for their potential role in the adaptation of rosemary. Many genes of the CYP family are involved in the biosynthesis of plant secondary metabolites (Bak et al., 2011). A total of 375 CYP genes were identified in the rosemary genome, and 17 CYP gene families with a total of 160 genes were expanded compared with other species. It contains eight CYP76A genes involved in carnosic acid biosynthesis (CYP76AK6, CYP76AK7 and CYP76AK8), eight CYP98A genes involved in rosmarinic acid biosynthesis, and two CYP93B genes involved in the apigenin and luteolin biosynthesis pathway (Figure 3a). The expansion of these gene families was conducive to the biosynthesis of antioxidant components in rosemary and enhanced its ability to resist adverse environment.

Abiotic stresses could lead to excessive reactive oxygen species (ROS) in plants. The peroxidase system plays an important role in the ROS homeostasis of plants and their adaptation to various abiotic stresses. A total of 118 peroxidases had been identified in the genome of rosemary, among which five families were expanded, including 17 peroxidase genes (Figure 3f), which could enhance the ability of rosemary to regulate ROS homeostasis and improve its stress resistance.

Heat shock protein (HSP) and heat stress transcription factor (HSF) were extremely important in response to environmental stresses by regulating protein homeostasis (Zhang *et al*., 2019). A total of 166 HSP genes were identified in the rosemary genome, while 24 out of 38 HSP70 genes were expanded. Forty‐six HSP90 genes were identified in the rosemary genome, and 45 of them were expanded. Meanwhile, three HSF gene families were expanded (Figure 3a–c). These results indicate that gene expansion of HSP and HSF may enhance the ability of protein homeostasis regulation, which could be an important strategy for rosemary's adaptation to unfavourable environments.

Ion transporters are necessary for plants to resist the ion imbalance caused by abiotic stresses, such as salt, heat and drought stresses. Potassium ion homeostasis in plants is realized by potassium ion transporters. In the rosemary genome, a total of 35 potassium transporters were identified; of which 27 genes were expanded (Figure 3e). The expansion of these gene families may enhance the ability of rosemary's ion homeostasis regulation and improve its adaptability to various stresses.

### Tissue‐specific distribution of antioxidant component and its potential role in environmental adaptation

**Figure 4:**
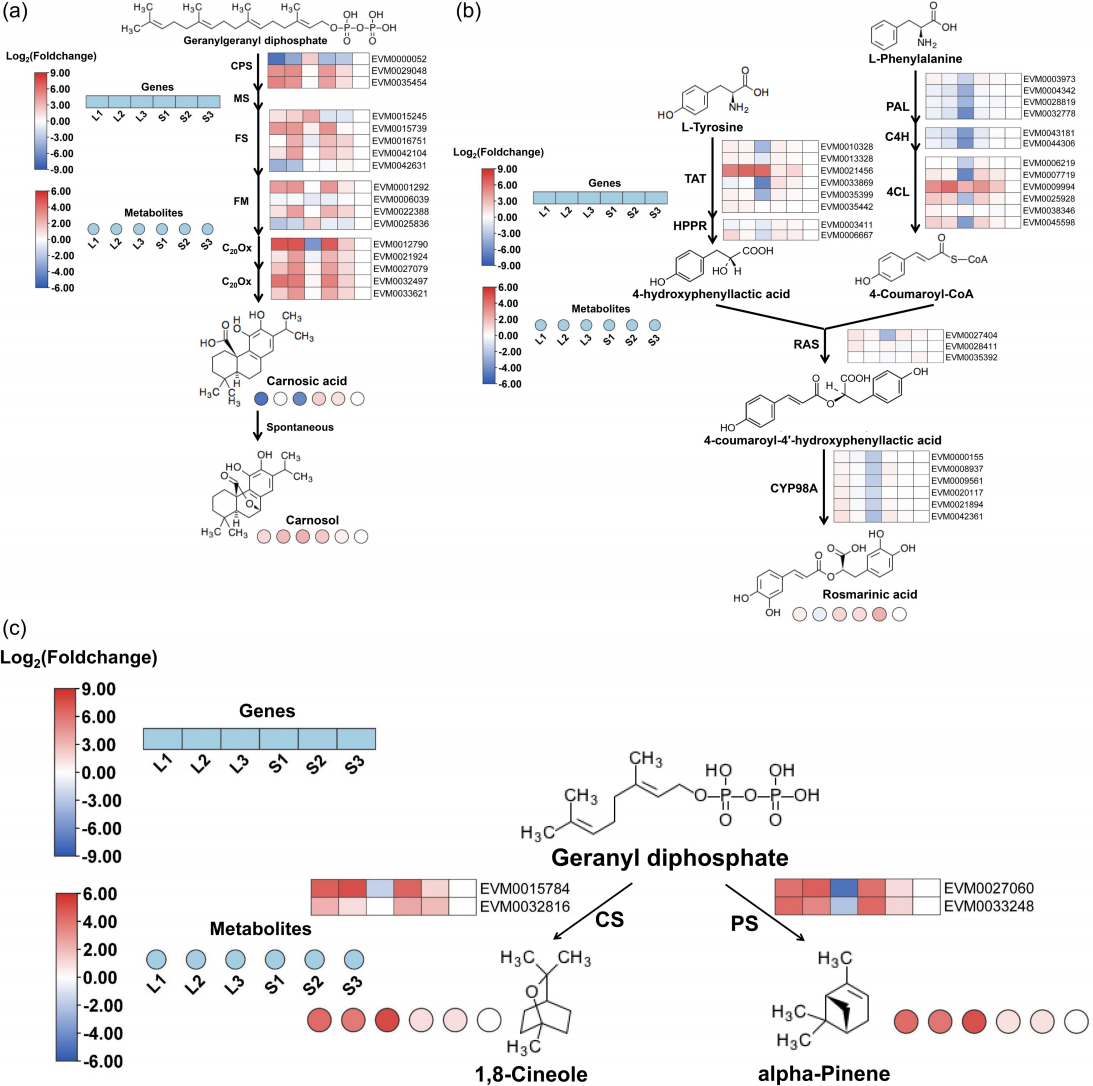
The biosynthetic pathway of main antioxidant components in rosemary. The biosynthetic pathway of carnosic acid, carnosol (a), rosmarinic acid (b) and the main volatile compounds (c). CPS, copalyl diphosphate synthase; MS, miltiradiene synthase; FS, ferruginol synthase; FM, ferruginol monooxygenase; C_20_Ox, C_20_‐oxidase (CYP76A); TAT, tyrosine aminotransferase; HRRP, hydroxyphenylpyruvate reductase; PAL, phenylalanine ammonia‐lyase; C4H, cinnamate 4‐hydroxylase (CYP73A); RAS, rosmarinic acid synthase; 3/3′‐hydroxylase (CYP98A); CS, cineole; PS, pinene synthase. L1, L2 and L3 represent 1 month, 6 months and 12 months leaves at 1‐year‐old shoot. S1, S2 and S3 represent shoots of three different lignification degrees determined by Fourier transform infrared spectroscopy.

In order to well understand the importance of antioxidant components in rosemary's adaptation to the environments, metabolomic and transcriptomic analyses were performed. Metabolomic analysis shows that antioxidant components were rich in rosemary leaves and shoots. Many non‐volatile antioxidant components, including fat‐soluble (carnosic acid, carnophenolic, oleanolic acid, ursolic acid, etc.) and water‐soluble antioxidant components (rosmarinic acid and various flavonoids, etc.), were also identified. Among them, carnosic acid, carnophenolic, rosmarinic acid and oleanolic acid have strong antioxidant capacity and are of high content in rosemary leaves and shoots (Ribeiro‐Santos *et al*., 2015). Carnosic acid was produced in the diterpene biosynthesis pathway, and geranylgeranyl diphosphate was mainly derived from the terpenoid backbone biosynthesis pathway, which was spontaneously converted to carnosol (Figure 4a) (Ignea *et al*., 2016; Scheler *et al*., 2016). Rosmarinic acid biosynthesis has been completely resolved and is derived from the metabolic pathways of tyrosine and phenylalanine metabolism (Figure 4b) (Petersen, 2013). Major volatile antioxidant components of rosemary (mainly including pinene, verbenone and cineole) were produced in monoterpenoid biosynthesis pathway, and the substrate geranyl diphosphate was mainly derived from the terpenoid backbone biosynthesis pathway (Figure 4c).

Metabolomic analysis shows that the contents of antioxidant components varied among different tissues of rosemary. The content of antioxidant components was higher in young tissues than that in the relatively mature tissues (Figure 4; Data Set S4 and Figure S4). Among the six tested tissues, carnosic acid content was the highest in S2 shoots and correspondingly carnosol in L1 leaves. The contents of six volatile active substances were mainly found in leaves but less in shoots, the highest contents of α‐pinene, cineole, borneol and verbenone in L3 leaves, correspondingly camphene and bornyl acetate in L1 leaves. In addition, 48 flavonoids, such as luteolin and saurin, were also detected in different tissues of rosemary, and their contents also varied among different tissues (Data Set S4 and Figure S4). Young tissues are generally more sensitive to environmental stresses than mature tissues (Huo *et al*., 2021; Munns and Tester, 2008; Rui *et al*., 2016; Sousa *et al*., 2021; Wang *et al*., 2012). These results indicate that more antioxidant components may contribute to rosemary's resistance to adverse environment.

Comparative transcriptome analysis among different tissues shows that differentially expressed genes (DEGs) were mainly involved in metabolic and oxidation–reduction processes (Data Sets S5 and S6). The KEGG (Kyoto Encyclopedia of Genes and Genomes) enrichment results shows that a number of DEGs were mainly involved in the biosynthetic pathways of phenylpropane, phenylalanine, tyrosine, tryptophan and flavonoids (Data Set S7), in which the main antioxidant components were generated. In addition, the biosynthesis pathway of terpenoids forming GPP is the basis of the biosynthesis of rosemary aromatic components. Furthermore, some genes in the GPP biosynthesis pathway were differentially expressed in different tissues, which also supported the key role of GPP in the biosynthesis of rosemary aromatic components. These results were consistent with the results of metabolome analysis. The combined transcriptome and metabolome analysis indicate that regulating the content of antioxidant components may play important roles in response to adverse environmental factors.

### Antioxidant substances involved in response and adaptation to environmental stresses revealed by comparative transcriptome

**Figure 5:**
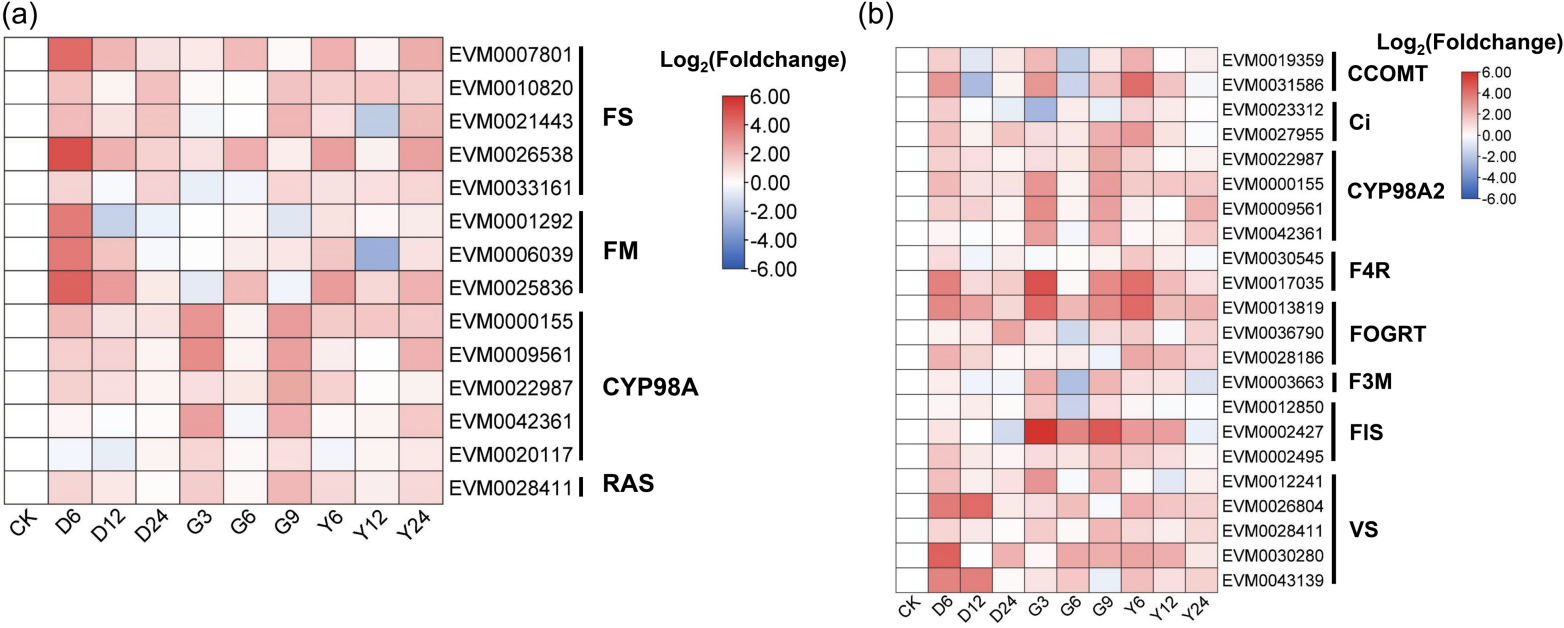
Expression patterns of key genes involved in response to the drought, heat and salt stresses. (a) DEGs in the biosynthesis pathway of carnosic acid and rosmarinic acid; (b) DEGs in the biosynthesis pathway of flavonoids. FS, ferruginol synthase; FM, ferruginol monooxygenase; CYP, cytochrome P450; RAS, rosmarinic acid synthase; CCOMT, caffeoyl‐CoA O‐methyltransferase; Ci, chalcone isomerase; CYP, cytochrome P450; F4R, flavanone 4‐reductase; FOGRT, flavanone 7‐O‐glucoside 2″‐O‐beta‐L‐rhamnosyltransferase; F3M, flavonoid 3′‐monooxygenase; FlS, flavonol synthase; VS, Vinorine synthase; D6, D12 and D24 represent drought stress for 6 h, 12 h and 24 h, respectively; G3, G6 and G9 represent heat stress for 6 h, 12 h and 24 h, respectively; Y6, Y12 and Y24 represent salt stress for 6 h, 12 h and 24 h, respectively.

**Figure 6:**
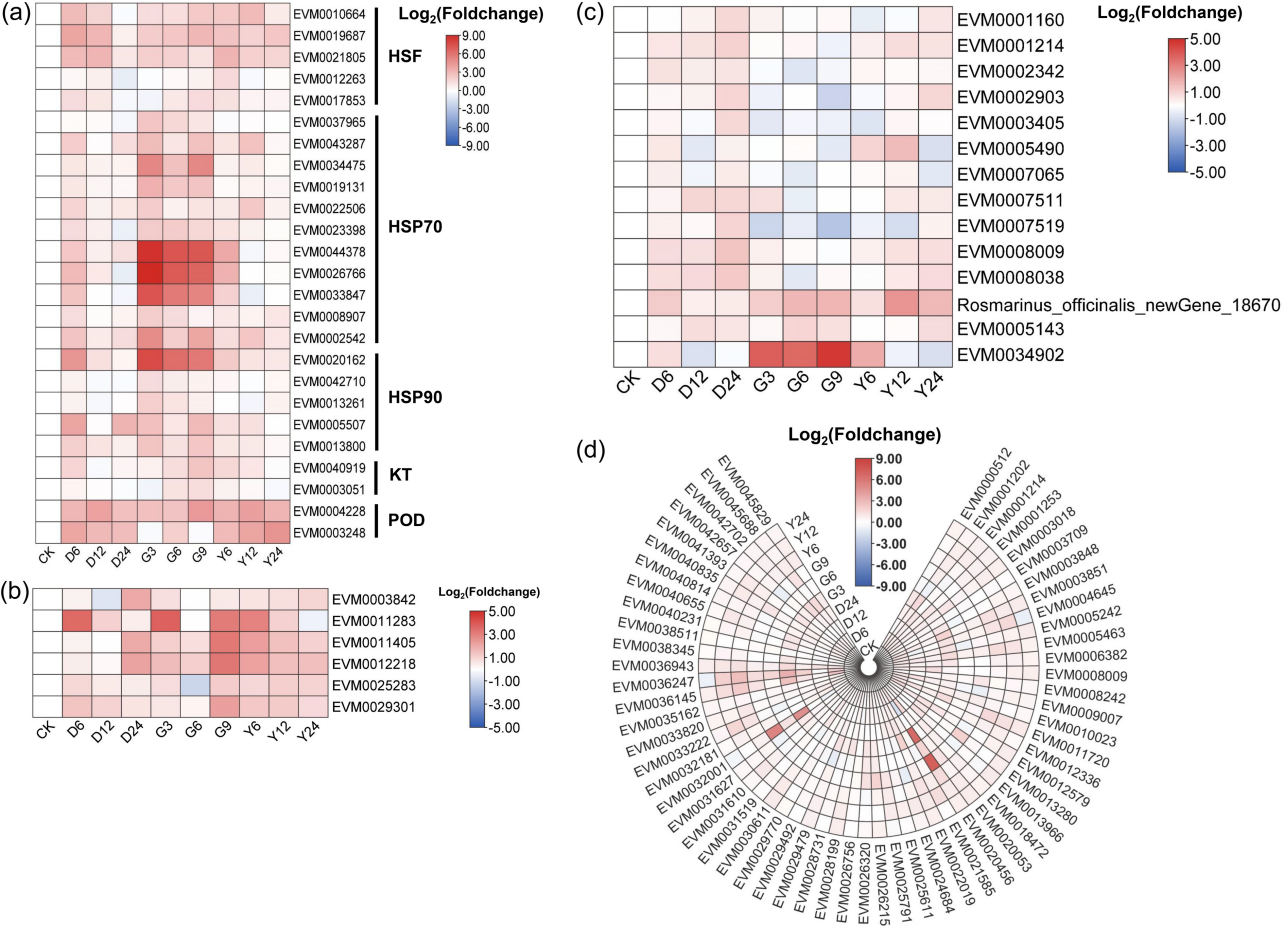
Expression patterns of key genes involved in response to the drought, heat and salt stresses. (a) Gene expression patterns of expanded HSF, HSP70, HSP90, KT and POD; (b) aquaporin gene expression patterns in response to three kinds of stress; (c) expression patterns of genes involved in photosynthesis; (d) expression patterns of ATP synthase genes in response to stresses. HSF, heat stress transcription factor; HSP, heat shock protein; KT, potassium transporter; POD, peroxidase; CK, leaves of plant under normal condition; D6, D12 and D24 represent drought stress for 6 h, 12 h and 24 h, respectively; G3, G6 and G9 represent heat stress for 6 h, 12 h and 24 h, respectively; Y6, Y12 and Y24 represent salt stress for 6 h, 12 h and 24 h, respectively.

Based on the metabolomic and transcriptomic analyses of different rosemary tissues, we further performed comparative transcriptome analyses of leaves under different environmental stresses, including drought, heat and salt, to investigate the role of antioxidant components in rosemary response to environmental stresses. Under short‐term drought, heat or salt stresses, a large number of DEGs are involved in metabolic processes (Data Sets S8–S11). Certain genes in the biosynthesis pathways of carnosic acid, rosmarinic acid and flavonoids were up‐regulated in all three tested environmental stresses (Figure 5), illustrating that these antioxidant components could be crucial in responding to abiotic stresses, by adjusting the ROS homeostasis, so as to maintain the stability of the membrane system, nucleic acid and metabolism, and to keep electrolyte and protein homeostasis (Hasanuzzaman *et al*., 2021; Jogawat *et al*., 2021; Kaushik and Aryadeep, 2014; Loussouarn *et al*., 2017). In addition, two of the expanded peroxidase genes (EVM0004228 and EVM0003248) were also up‐regulated under the three stresses (Figure 6a), which may be further enhanced the rosemary's ability to maintain ROS homeostasis. These up‐regulated genes may enhance the biosynthesis of antioxidant components in rosemary, and then contribute to the regulation of ROS homeostasis, suggesting the importance of ROS homeostasis in rosemary's adaptation to various abiotic stresses.

### Molecular model of rosemary adaptation to abiotic stresses

In addition to genes involved in the biosynthesis of antioxidant components, other expanded gene families related to environmental stresses were also analysed. Among the HSP gene families expanded in rosemary genome, 11 HSP70 genes such as EVM0037965, EVM0043287 and EVM0034475 were mainly up‐regulated in the three stress environments. Five HSP90, including EVM0020162, EVM0042710 and EVM0013261, were also up‐regulated in the three stress environments (Figure 6a). Additionally, five expanded HSF genes were also up‐regulated. These results indicated that these HSP and HSF genes played an important role in maintaining protein homeostasis in rosemary to respond to adverse environments. Among the potassium ion transporters that expanded, two were up‐regulated under the three stresses, potentially helping rosemary maintain ion homeostasis in cells (Figure 6a).

Drought, heat and salt stresses affected water absorption of plants, resulting in water imbalance. Aquaporins play an important role in regulating osmotic pressure and maintaining water homeostasis in plants. After rosemary exposure to a short period of stresses, six AQPs, such as EVM0003482, were significantly up‐regulated to maintain water homeostasis and respond to drought, heat or salt stress (Figure 6b).

Under stress treatments, 14 genes involved in rosemary photosynthesis were partially up‐regulated, especially the photosystem II oxygen‐evolving enhancer protein gene PsbP under heat stress (Figure 6c). These genes were not significantly affected by various stresses, so as to stabilize the photosynthetic efficiency and provide materials and energy for rosemary's resistance to various stresses. The expression of 60 ATP synthase genes was also not significantly affected by these three stresses, and some were up‐regulated, which provided energy for rosemary's response to various stresses (Figure 6d).

**Figure 7:**
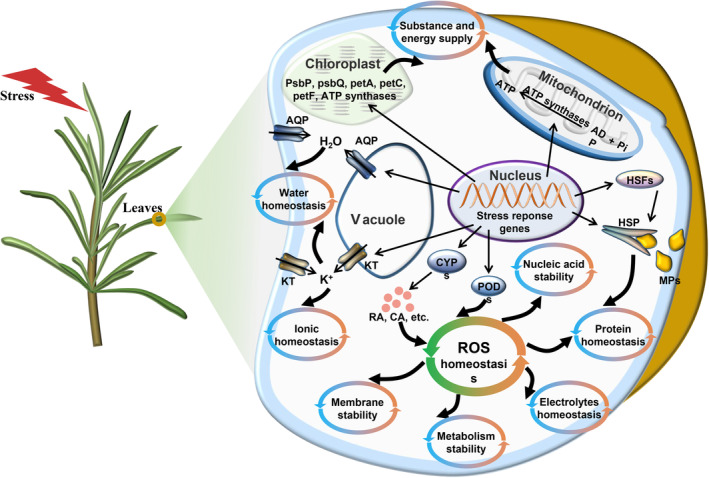
Molecular model of rosemary adaptation to drought, heat and salt stresses. Under short‐term stresses, rosemary can regulate the ROS homeostasis by increasing the biosynthesis of antioxidant components, improve the photosynthetic efficiency, maintain capacity supply and provide material and energy for adapting to stresses. To maintain protein balance, ionic balance and other systematic balance, and finally improve the ability of rosemary to resist stresses. AQP, aquaporin; CYP, cytochrome P450; HSP, heat shock protein; HSF, heat stress transcription factor; KT, potassium transporter, PsbP, photosystem II oxygen‐evolving enhancer protein 2; PsbQ, photosystem II oxygen‐evolving enhancer protein 3; PetA, apocytochrome f; petC, cytochrome b6‐f complex iron–sulphur subunit; petF, ferredoxin; POD, peroxidase; RA, rosmarinic acid; SA, carnosic acid; MP, misfolded protein.

Generally, rosemary combines antioxidant components and POD to maintain the ROS homeostasis in cells, which results in maintaining the stability of the membrane system, nucleic acid and metabolism cells, keeps the balance of electrolyte and protein, and further cooperates with photosynthesis, energy metabolism, ion and water homeostasis mechanism, to adapt to the changing environments (Figure 7).

## Discussion

High‐quality genomic sequence information is the foundation for both gene discovery and crop improvement. This study used the Nanopore sequencing technology and Hi‐C‐assisted assembly technology to obtain a chromosome‐level genome of rosemary. A draft genome (cultivar Arp) assembled by Nolan *et al*. (2020) and a chromosome‐level genome (cultivar Morocco) assembled by Han *et al*. (2023) are 1.01 Gb and 1.11 Gb, respectively, while here a 1.20 Gb chromosome‐level genome (cultivar Albus‐2) was assembled. The scaffold N50 of the three genomes is 368.74 kb, 95.5 Mb and 87.94 Mb, respectively. Moreover, 43 859 genes were functionally annotated in this study, 3158 more than those annotated by Han et al. Importantly, based on the comparison between Albus‐2 and Morocco genomes, a total of 16 114 187 genetic variations were identified (Figure S5 and Table S3). This suggested that there could be many genetic variants between the two accessions, Albus‐2 and Morocco, which may provide important information on cultivar divergence and identifying more unique genes for crop improvement. In the future, the telomere‐to‐telomere (T2T) gap‐free genome of rosemary could be obtained based on the Nanopore ultra‐long (N50 > 100 kb) technology, combined with PacBio HiFi. The assembly of more genomes for different cultivars to construct the pan‐genome of rosemary will provide important resources for gene discovery and breeding.

At the same time, we also employed comparative genome, transcriptome and metabolism to investigate the molecular mechanism of rosemary in response to changeable environments and showed the importance of antioxidant components in rosemary's adoption to the harsh environment. The role of antioxidant components in plant response to adverse environments has always been an important perspective for investigating the mechanism of plant adaptation, and the specific molecular mechanism is rather complex (Loiacono and Tullio, 2012). However, the mechanism and effect of many small antioxidant components such as carnosic acid, rosmarinic acid, oleanolic acid and ursolic acid are still unclear in plant adaptation. It has been revealed that carnosic acid, rather than carnosol, is a ROS chemical quencher *in vitro* analyses, which protects lipids from oxidation (Loussouarn *et al*., 2017). The regulation of ROS homeostasis plays an important role in plant response to abiotic stresses (You and Chan, 2015). This study further indicates that the main antioxidant components are involved in rosemary's response to various environmental stresses (drought, heat and salinity), which may play an important role by regulating ROS homeostasis, but the specific regulatory mechanism and the effect of antioxidant components in plant resistance to abiotic stresses still need to be further studied.

Many studies show that young tissues are more sensitive to environmental stress than mature tissues (Huo *et al*., 2021; Munns and Tester, 2008; Rui *et al*., 2016; Sousa *et al*., 2021; Wang *et al*., 2012). Secondary metabolites, such as phenolic acids, terpenoids and flavonoids, play important roles in plant adaptation to the harsh environment (Kessler and Kalske, 2018; Liu, Luo *et al*., 2021; Nakabayashi *et al*., 2014; Pang *et al*., 2021). In response to adverse conditions, a large number of secondary metabolites are synthesized in young plant tissues (Mishra *et al*., 2021). This suggests that young tissues with more synthesized secondary metabolites may help them respond to various stresses. In this study, we found that the content of carnosic acid was much higher in young leaves (16.9‐fold) than that in mature leaves (L3) (Figure 4a; Data Set S4). Ursolic acid contents were more than 100% higher in L1 and L2 leaves than in L3 leaves, in S1 and S2, which were 13.9‐fold and 8.4‐fold of that in S3, respectively (Data Set S4). Linoleic acid contents in L1 and L2 were 1.5‐fold and 1.6‐fold of that in L3, in S1 and S2, which were 5.4‐fold and 4.0‐fold of that in S3, respectively (Data Set S4). The results also show that the contents of some antioxidant components were higher in young tissues than that in mature tissues, suggesting that the antioxidant components may play an important role in rosemary's response to abiotic stresses.

Rosemary originates from the Mediterranean basin, where plants are often subject to various abiotic stresses, especially in summer, such as drought, heat, strong light and salinity (Munné‐Bosch and Alegre, 2000). The leathery and linear characteristics of rosemary leaves help rosemary resist various stresses. Rosemary also synthesizes various antioxidant components, which may act as small‐molecule ROS scavengers to regulate ROS homeostasis and then enhance rosemary's resistance to abiotic stresses (Apel and Hirt, 2004; Hasanuzzaman *et al*., 2021; Jogawat *et al*., 2021; Kaushik and Aryadeep, 2014; Murphy *et al*., 2011) (Figure 7). It has been elucidated that CYP76AK6–8 genes code C_20_ oxidases, which sequentially catalyse the conversion of 11‐hydroxyferruginol to carnosic acid (Guo *et al*., 2013; Ignea *et al*., 2016). The final biosynthetic steps of rosmarinic acid are the 3‐ and 3′‐hydroxylation of 4‐hydroxycinnamoyl‐4′‐hydroxyphenyllactate catalysed by cytochrome P450 enzymes of the CYP98A family (Petersen, 2013; Schoch *et al*., 2018). In rosemary genome, some CYP gene families, such as CYP76A and CYP98A, were expanded to facilitate the synthesis of antioxidant components and reduce the damage of excessive ROS to cell structure and components. Moreover, after short period of stress treatments, certain genes involved in carnosic acid, rosmarinic acid and flavonoids biosynthesis pathways were up‐regulated (Figure 5), probably resulting in synthesizing more antioxidant components to cope with excessive ROS. This also suggests that the rich in antioxidant components may be the result of the long‐term evolution of rosemary's adaptation to the environment. The key stress‐response hormone jasmonic acid (Wang *et al*., 2021) may be involved in carnosic acid synthesis (Han *et al*., 2023; Yao *et al*., 2022), but whether it regulates CYP76A remains to be further verified.

HSFs are the core transcription factors in plant response to heat stress and activate downstream response genes, which are modulated by calcium, ROS and ABA signalling (Jacob *et al*., 2017; Zhang *et al*., 2019, 2022). HSPs play a key role in conferring heat tolerance by maintaining proteins in their functional conformations and preventing the aggregation of non‐native proteins (Wang *et al*., 2004). Studies have also shown that HSP and HSF play an important role in plant resistance to various abiotic stresses such as drought, salt and cold stress (Jacob *et al*., 2017; Wang *et al*., 2004). In this study, we found that both HSF and HSP gene families were expanded in the rosemary genome, which may promote the expression of HSF and HSP genes under stress. Comparative transcriptome analysis showed that the expressions of certain HSF, HSP70 and HSP90 genes were also significantly up‐regulated in response to the three stresses (Figure 6a). The up‐regulated HSFs may enhance the expression of downstream genes associating with stress response. Under stress, HSP70 can prevent aggregation of denatured proteins and refold stress‐denatured proteins (Sung *et al*., 2001), which enhances the regulation of protein homeostasis and improves rosemary's tolerance. HSP90 can participate in refolding denatured proteins to maintain conformation of numerous proteins, and also mediate extensive stress signal transduction (Wang *et al*., 2004). Whether these genes or other members of the families collaborate to participate in the protein homeostasis system under multiple stresses needs further study.

Abiotic stresses often cause water imbalance in plants. Aquaporins are membrane channels that facilitate the transport of water and small neutral molecules across biological membranes, which play an important role in regulating water homeostasis in cells (Maurel *et al*., 2015; Törnroth‐Horsefield *et al*., 2006). Studies have shown that under various abiotic stresses, plants regulate the expression of aquaporins to promote their resistance (Aroca *et al*., 2012; Wang *et al*., 2014). In this study, six aquaporin genes were significantly up‐regulated during short period of heat, drought or salinity stresses (Figures 6b and 7), including three plasma membrane intrinsic protein genes, one tonoplast intrinsic proteins gene and two small basic intrinsic proteins genes. These aquaporins may play a positive regulatory role in rosemary's response to stresses. Moreover, some aquaporins could contribute to plant resistance to multiple abiotic stresses (Maurel *et al*., 2015; Wang *et al*., 2014). These six genes could also cooperatively regulate water transport in rosemary under multiple abiotic stresses to maintain the water homeostasis in cells, which may enable plants to recover quickly after stresses. The cooperative mechanism of them in regulating water homeostasis is worthy of further study.

In general, the mechanism of rosemary's adaptation to the environment is rather complex, and the homeostasis of ROS, proteins and water is essential in its response to various stresses, while more insights into the specific regulatory mechanism are needed. In the future, gene functional identification and transcription regulation research will be conducted to well reveal the molecular regulatory mechanisms of these key genes in rosemary's adaptation to various stresses.

## Conclusion

Based on the third‐generation high‐throughput sequencing and Hi‐C technologies, we sequenced and assembled a high‐quality chromosome‐level genome for a novel rosemary cultivar (Albus‐2) with high tolerance to environmental stresses. Combined with transcriptome and metabolic analysis, we revealed the molecular basis of the differential antioxidant component distribution in different rosemary tissues. Under abiotic stresses, 36 genes involved in the biosynthesis of carnosic acid, rosmarinic acid and flavonoids were up‐regulated, which play an important role in rosemary's response to various stresses. A molecular model on rosemary's adaptation to harsh environments was proposed, and the regulatory mechanism of antioxidant components against stress was addressed. This study provides a new genomic data platform for gene discovery and breeding in rosemary, and also provides a new insight into the accumulation of antioxidant components and environmental adaptation of rosemary.

## Materials and methods

### Materials

Based on screening resistance to various stresses, the line ‘Albus‐2’ was selected from the sexual progeny of the main rosemary cultivar ‘Albus’ in China, which is high resistant to drought, heat and salt stresses. The plants were grown in the Greenhouse of Henan Agricultural University. Fresh leaves (grown 6 months on 1‐year‐old shoot) were collected and immediately frozen in liquid nitrogen and then stored in the −80 °C freezer.

### DNA library construction and sequencing

High‐quality genomic DNA (gDNA) was extracted from the leaves using the CTAB method (Chang *et al*., 1993). At least 15 μg gDNAs with high quality (no impurity and degradation) were used and randomly broken into small pieces, and then were enriched and purified by magnetic beads and larger than 15 kb fragments were separated through agarose gel electrophoresis. These fragments were repaired by damage repair, end repair and adding A to the 3′ end, and then were purified. The repaired fragment products were connected with sequencing adapters and purified to obtain the final library. Qubit 3 (Thermo Fisher Scientific, Waltham, MA, USA) was used to quantitatively detect the built DNA library. Real‐time single‐molecule sequencing was performed on PromethION sequencer (Oxford Nanopore Technologies PLC, Oxford, UK) to obtain raw sequencing data using Guppy in the MinKNOW package with default parameters (Wick *et al*., 2019). Clean data were obtained after filtering out adapters and low‐quality reads.

### Genome assembly

Clean data were corrected with Canu software based on the falcon_sense method (Corrected ErrorRate = 0.025) (Koren *et al*., 2017) and then were assembled by WTDBG (Ruan and Li, 2020) and SMARTdenovo (Liu, Wu *et al*., 2021), respectively, with default parameters. The assembly results of the two software were integrated by quickmerge (Chakraborty *et al*., 2016) to improve assembly quality, and the third‐generation sequencing data were polished three times by Racon (Vaser *et al*., 2017), and then, contigs were polished three times through Pilon (Walker *et al*., 2014) with default parameters.

In order to assist in assembling the chromosome of the genome, we collected rosemary leaves for Hi‐C sequencing. DNA was cross‐linked with formaldehyde after nuclear integrity detection and then digested with *Hind* III. The obtained fragment was used for the end repair mechanism. Biotin‐labelled bases were introduced, and the repaired DNA was cycled. Then, it was broken into 300–700 bp fragments, using strand avidin magnetic beads to capture DNA fragments containing interaction relations for library construction. Qubit 3 (Thermo Fisher Scientific, Waltham, MA, USA) and Agilent Bioanalyzer 2100 system (Agilent, Santa Clara, CA, USA) were used to detect the concentration and insert size of the library, respectively. The effective concentration of the library was accurately quantified by qRT‐PCR to ensure the quality of the library. Illumina HiSeq X Ten Platform was used for sequencing. Hi‐C read pairs were submitted to LACHESIS Software (Burton *et al*., 2013) to refine the genome assembly, which would result in chromosome‐length scaffolds. Hi‐C was used to assist the assembly, and PbJelly (English *et al*., 2012) was used to fill gap after Hi‐C assembly, and the fine genome map of rosemary was finally obtained.

Genomic Illumina reads and the RNA‐seq reads were remapped to the final genome assembly to assess the reads alignment rate. The completeness and accuracy of genome assembly were quantitatively assessed by the BUSCO (ver.5.2.1, Simao *et al*., 2015).

### Genome annotation

Since the conservation of repeats among species is relatively low, it is necessary to construct specific repeats database when predicting repeats for a specific species (Kutil and Williams, 2001; Price *et al*., 2005). Therefore, with LTR_FINDER (Xu and Wang, 2007) and RepeatScout (Price *et al*., 2005), we constructed the repeat sequence database of the genome based on the principle of structure prediction and *de novo* prediction. PASTEClassifier (Hoede *et al*., 2014) was used to classify the database and then combined with the Repbase database (Jurka *et al*., 2005) as the final repeat sequence database. Then, RepeatMasker software (Tarailo‐Graovac and Chen, 2009) was used to predict the repeat sequences of the genome based on the constructed repeat sequence database.

Three different strategies were used to predict the gene structure of the genome. Genscan (Burge and Karlin, 1997), Augustus (Stanke and Waack, 2003), GlimmerHMM (Majoros *et al*., 2004), GeneID (Blanco *et al*., 2007) and SNAP (Korf, 2004) were used for *de novo* prediction. GeMoMa (Keilwagen *et al*., 2016, 2018) for homologous species prediction. Based on transcriptome data, gene prediction was performed by TransDecoder, GeneMarkS‐T (Tang *et al*., 2015) and PASA (Campbell *et al*., 2006). Finally, EVM (Haas *et al*., 2008) was used to integrate the prediction results obtained by the above three methods, and PASA was used to modify them. Default parameters were used for all programs. The predicted gene sequences were compared with NR (ftp://ftp.ncbi.nih.gov/blast/db), KOG (ftp://ftp.ncbi.nih.gov/pub/COG/KOG/kyva), GO (http://geneontology.org), KEGG (http://www.genome.jp/kegg) and TrEMBL (subdatabase of UniProt, https://www.uniprot.org/) by BLAST V2.2.31 (*E*‐value ≤1e‐5).

Genome‐wide alignment was performed to identify rRNA and microRNA using Blastn based on Rfam database (Griffiths‐Jones *et al*., 2005). tRNA was identified by TrnasCAN‐SE (Lowe and Eddy, 1997) with default parameters. Using the predicted protein sequences, we searched for homologous gene sequences (possible genes) on the genome by GenBlastA (Rong *et al*., 2009) and then by GeneWise (Birney *et al*., 2004) to search for premature stop codons or frameshift mutations in the gene sequences to obtain pseudogenes.

### Comparative genomics analysis

Protein sequences of *Rosmarinus officinalis* and 11 other species (*S. Splendens, S. miltiorrhiza, S. indicum, O. europaea, S. lycopersicum, A. Trichopoda, O. siativa, A. Thaliana, B. vulgaris, G. ax, V. Vinifera*) were extracted from the public database (UniProt and Phytozome). Based on the sequence alignment results, the sequences and structures of known genes were compared. The duplication of genes within species, the evolution among species and the classification of species‐specific genes were analysed. Family classification of protein sequences of each species was carried out by OrthoMCL software (Li *et al*., 2003) to find the unique gene families of rosemary. We used PHYML software (Guindon *et al*., 2010) to construct an evolutionary tree based on the single‐copy protein sequences of rosemary and 11 other species to study the evolutionary relationship between species. We selected a certain fossil calibration time and used Mcmctree (Puttick, 2019) to estimate the differentiation time between species. Gene family contraction and expansion was performed using (De Bie *et al*., 2006). Based on the Branch model, the CodeML module of PAML (Bielawski *et al*., 2016) with default parameters was used to analyse the selection pressure of single‐copy genes of each species, and genes under positive selection were determined by likelihood ratio tests (*P* < 0.05) and Bayes empirical Bayes method (posterior probability >0.95), and then, the KOG, GO and KEGG annotation were performed with the clusterProfiler. R package (Wu *et al*., 2021) was used for enrichment analysis. The 4DTv and synonymous substitution rate (Ks) were applied to detect WGD events. MCscan (Wang *et al*., 2013) was applied to detect the 4DTV gene pairs calculated using the HKY substitution model. MCscan with the YN00 program of the PAML package (Yang, 2007) was used to detect Ks of gene pairs.

### Metabolome analysis

Leaves at three stages (L1, L2 and L3 represent 1 month, 6 months and 12 months leaves at 1‐year‐old shoot) and shoots at three different lignification degrees (S1, S2 and S3 determined by Fourier transform infrared spectroscopy, FTIR) (Nicolet iS10, Thermo Fisher Scientific, Waltham, MA, USA) (Figure S6) were selected to use in this study. There were a total of 18 samples in three biological replicates. Water‐soluble metabolites were extracted by water extraction, and the metabolites were determined by UHPLC–MS/MS (Suzuki *et al*., 2022). For UHPLC–MS/MS assay, the Vanquish Flex UHPLC system was coupled to Q Exactive Plus mass spectrometry (Thermo Fisher Scientific, Rockford, IL, USA) for metabolite separation and detection. The MS data acquisition was performed by Q Exactive Plus (Thermo Fisher Scientific, Rockford, IL, USA) system. The lipid‐soluble antioxidant components (carnosic acid, ursolic acid and oleanolic acid) were extracted with 70% ethanol and determined by ultra‐performance liquid chromatography‐triple quadrupole tandem mass spectrometer (Mena *et al*., 2016). Samples were analysed using the Xevo TQ‐XS system (Waters, USA) equipped with an ESI ion source. Volatile metabolites were determined by GC–MS, used an Agilent Technologies 7890B gas chromatogram (Agilent, Santa Clara, Calif., USA) coupled to a Xevo TQ‐GC mass detector (Waters, Milford, NE, USA). Between two samples, differentially expressed metabolites were determined with a fold‐change cut‐off of ≥1.2 or ≤0.83, *P*‐value <0.05, and variable importance in projection (VIP) ≥ 1.0. The difference in metabolite content between samples was compared to determine the biosynthesis pathway of the different metabolites. A supplementary file for the detection of water‐soluble and volatile metabolites with more details was supplied in the Source Data file (Method S1).

### Comparative transcriptome analysis in different tissues

The samples were collected in the same way as metabolome analysis, and then, RNA‐seq analysis was performed. Total RNAs were isolated from 1 g sample using the TRIzol reagent (Invitrogen, 15596026) according to the manufacturer's instructions. RNA concentration and purity were measured using NanoDrop 2000 (Thermo Fisher Scientific, Wilmington, DE). RNA integrity was assessed using the RNA Nano 6000 Assay Kit of the Agilent Bioanalyzer 2100 system (Agilent Technologies, CA, USA). A total of 1 μg RNAs per sample were prepared for constructing RNA‐seq libraries, which were generated using NEBNext^®^ UltraTM RNA Library Prep Kit for Illumina^®^ (NEB, USA) according to the manufacturer's recommendations. The PCR was performed with Phusion high‐fidelity DNA polymerase, and library quality was assessed on an Agilent Bioanalyzer 2100 system (Agilent, Santa Clara, CA, USA). The libraries were sequenced on an Illumina NovaSeq 6000 platform. Clean reads were obtained by removing reads containing adapters, reads containing poly‐N and low‐quality reads from raw data. Paired‐end clean reads were aligned to the reference genome using Hisat2 tools (Kim *et al*., 2015). The featureCounts (Liao *et al*., 2014) was used to count the read numbers mapped to each gene. Then, gene expression levels were estimated by fragments per kilobase of transcript per million fragments (FPKM) mapped. Genes with FPKM >1.0 were defined as being expressed. DEGs were detected by DESeq (Love *et al*., 2014). The Benjamini–Hochberg method was used to control the false discovery rate (FDR). Genes with an FDR <0.01 and Fold Change ≥2 were assigned as DEGs. GO and KEGG enrichment analysis of DEGs were implemented by the GOseq R package (Young *et al*., 2010) and KOBAS software (Xie *et al*., 2011).

### Comparative transcriptome analysis for plant response to stresses

In order to further study the molecular response of rosemary to drought, heat and salt stresses, the leaves growing normally and under the three stresses were collected for RNA‐seq and comparative transcriptome analysis. The 15‐cm cutting seedling with the same growth status was selected for drought, heat and salt stresses. Plants were treated with 20% PEG6000 for drought experiment, sampling at 0, 6, 12 and 24 h after treatments. Heat stress experiment was performed in the light incubator. The conditions were 40 °C with 12/12 light/dark, sampling at 0, 3, 6 and 9 h after treatments. In the salt stress experiment, plants were treated with 200 mM NaCl, sampling at 0, 6, 12 and 24 h. Three biological replicates were performed for each treatment. RNA‐seq and bioinformatics analysis were performed as described previously.

### Quantitative real‐time PCR validation

Quantitative real time PCR (qRT‐PCR) analysis was used to verify RNA‐seq results based on 10 randomly selected genes involved in antioxidant component biosynthesis. The primers of these 10 genes are listed in Table S4. The RNA samples used for qRT‐PCR assays were the same as those used for RNA‐seq. The qRT‐PCR was performed with SYBR^®^ PremixDimerEraser™ (Takara, Dalian, China) according to the manufacturer's specifications. The reaction mixtures were incubated at 95 °C for 3 min, followed by 40 cycles of 95 °C for 5 s, 64 °C for 60 s and 72 °C for 30 s. Rosemary eIF gene was used as a reference to normalize the amount of gene‐specific qRT‐PCR products (Scheler *et al*., 2016), whose stability was evaluated by an computational program RefFinder (Xie et al., 2023). The relative expression levels of selected genes were calculated with the 2^−ΔΔCt^ method (Schmittgen and Livak, 2008) (Figure S7 and Table S4). The Pearson correlation coefficient (*r*) was calculated to evaluate the concordance between RNA‐seq and qRT‐PCR results (Figure S8).

## Conflict of interest

These authors declare no competing interests.

## Author contributions

Mingwan Li, Baohong Zhang and Dangquan Zhang designed the experiments; Yong Lai, Jinghua Ma and Xuebin Zhang conducted the experiments and analysed the data; Xiaobo Xuan, Fengyun Zhu, Shen Ding, Turgay Unver, George Huo, Fude Shang and Yuanyuan Chen analysed the RNA‐seq data; Bing Zhao, Chen Lan, Ximei Li, Yihan Wang, Yufang Liu, Mengfei Lu and Deshuang Yang completed the metabolome analysis; Yong Lai wrote the manuscript; Turgay Unver, George Huo, Xiaoping Pan, Mingwan Li, Baohong Zhang and Dangquan Zhang revised the manuscript. All authors have read and approved the manuscript.

## Supporting information


**Data Set S1** Annotated genes in rosemary.

**Data Set S2** GO counts and enrichment of expanded genes in rosemary.

**Data Set S3** Counts and enrichment of positive selective genes in rosemary.

**Data Set S4** Metabolites determined in different rosemary leaves and shoots.

**Data Set S5** Statistic of comparative transcriptome for rosemary leaves and shoots at different stages.

**Data Set S6** GO counts and enrichment of DEGs for rosemary leaves and shoots at different stages.

**Data Set S7** KEGG counts and enrichment of DEGs for rosemary leaves and shoots at different stages.

**Data Set S8** Statistic of comparative transcriptome for rosemary leaves under heat, drought and salt stresses.

**Data Set S9** GO counts and enrichment of differentially expressed genes in rosemary leaves under drought, heat and salt stresses.

**Data Set S10** KEGG counts and enrichment of differentially expressed genes in rosemary leaves under drought, heat and salt stresses.

**Data Set S11** Genes involved in ROS homeostasis regulation responding to heat, drought and salt stresses.

**Figure S1** LTR insertion time of rosemary.
**Figure S2** GO annotation counts of the expanded genes in rosemary.
**Figure S3** GO annotation counts for positive selective genes in rosemary.
**Figure S4** The biosynthetic pathway of flavonoids (naringenin, apigenin and luteolin). CS, chalcone synthase; Ci, chalcone isomerase; CYP75A, flavonoid 3′,5′‐hydroxylase; CYP75B, flavonoid 3′‐monooxygenase; CYP93B, flavone synthase II.
**Figure S5** Synteny map and structural variation distribution between Albus‐2 and Morocco genomes. Reference represents chromosome of Albus‐2, and query represents chromosome of Morocco.
**Figure S6** FTIR spectra of rosemary shoots under different lignification degrees.
**Figure S7** The relative expression level of selected genes determined by qRT‐PCR analysis.
**Figure S8** The correlation between RNA‐seq and qRT‐PCR.
**Table S1** Basic statistical information of the rosemary genome sequencing and assembly.
**Table S2** Statistical information for gene families in rosemary.
**Table S3** Statistical information for genetic variations between Albus‐2 and Morocco.
**Table S4** Primers for qRT‐PCR analysis.

## Data Availability

The genome and transcriptome sequences described in this manuscript have been submitted to the National Center for Biotechnology Information (BioProject ID PRJNA830648 and BioSample accession SAMN27735633). The data underlying Figures 1, 2, 3, 4, 5, 6, 7 and Figures S1–S8, as well as Table 1 and Tables S1–S5 and Data Sets [Link], [Link], are provided as a Source Data file.
