# Supplementary material for: High‐quality chromosome‐level genome assembly and multi‐omics analysis of rosemary (Salvia rosmarinus) reveals new insights into the environmental and genome adaptation
Source: Plant Biotechnol J. 2024 Feb 16;22(7):1833–47. doi: 10.1111/pbi.14305 (PMC11182591; doi:10.1111/pbi.14305)
Supplement: Supplementary file 12 — Figure S1 LTR insertion time of rosemary. Figure S2 GO annotation counts of the expanded genes in rosemary. Figure S3 GO annotation counts for positive selective genes in rosemary. Figure S4 The biosynthetic pathway of flavonoids (naringenin, apigenin and luteolin). CS, chalcone synthase; Ci, chalcone isomerase; CYP75A, flavonoid 3′,5′‐hydroxylase; CYP75B, flavonoid 3′‐monooxygenase; CYP93B, flavone synthase II. Figure S5 Synteny map and structural variation distribution between Albus‐2 and Morocco genomes. Reference represents chromosome of Albus‐2, and query represents chromosome of Morocco. Figure S6 FTIR spectra of rosemary shoots under different lignification degrees. Figure S7 The relative expression level of selected genes determined by qRT‐PCR analysis. Figure S8 The correlation between RNA‐seq and qRT‐PCR. Table S1 Basic statistical information of the rosemary genome sequencing and assembly. Table S2 Statistical information for gene families in rosemary. Table S3 Statistical information for genetic variations between Albus‐2 and Morocco. Table S4 Primers for qRT‐PCR analysis. [file PBI-22-1833-s004.doc]

**Supplementary Methods**

**Water-soluble metabolites**

**Metabolites Extraction**

All collected samples were flash frozen and ground to a fine powder with a mortar and pestle. The powder of each sample was extracted overnight using 80% HPLC grade methanol containing 1 μM chrysin as the internal standard and the ratio of the fresh weight to the volume of extraction solution was kept as 0.1 g/mL. The undissolved sample residues were precipitated by centrifugation at 13000 rpm for 30 min at 4°C. The clear supernatants were loaded into injection vials and ready for UHPLC-MS/MS.

**High-Performance Liquid Chromatography**

For UHPLC-MS/MS assay, the vanquish-flex UHPLC system was coupled to Q Exactive Plus mass spectrometry (Thermo Fisher Scientific) for metabolite separation and detection. A Hypersil GOLD column (2.1×100 mm.1.9 μm; Thermo Fisher Scientific) was employed for compound separation at 30°C and 1 μL of sample was loaded. The mobile phase A was HPLC grade H_2_O with 0.1% (v/v) formic acid (Merck, Germany) and phase B was HPLC grade acetonitrile (Merck, Germany). The gradient elution conditions were set as follows: From 0 to 2 min, the mobile phase B increases to 10%; From 2 to 10 min, the mobile phase B increases to 50%; From 10 to 10.1 min, the mobile phase B increases to 80%; From 10.1 to 13 min, the mobile phase B was kept at 80%; From 13 to 14 min the mobile phase B increases to 95%; From 14 to 18 min the mobile phase B decreased to 10%. The flow rate was 0.3 ml/min.

The MS data acquisition was performed by Q Exactive Plus (ThermoFisher Scientific, Rockford, IL) system. In full scan MS/ddMS2 mode, the resolutions of full scan MS and ddMS2 were set at 70000 and 17500, respectively. The automatic gain control (AGC) target and maximum injection time in full scan MS settings were 1^e6^ and 100 ms, while their values were 2^e5^ and 50 ms in dd MS2 settings. The TopN (N, the number of top most abundant ions for fragmentation) was set to 8, and collision energy was set to 20%, 40% and 60%. A heated ESI source was used at positive and negative ion mode. The spray voltage was set as 3.5 KV for positive mode and 3.2 KV for negative mode. The capillary temperature and aux gas heater temperature were set as 320 and 350°C, respectively. Sheath gas and aux gas flow rate were set at 35 and 15 (in arbitrary units), respectively. The S-lens RF level was 50.

**Data Processing.**

Following LC−MS analysis, raw data were collected and processed using Compound Discoverer 3.2 (Thermo Fisher Scientific) with the metabolite databases including mzCloud, mzVault, Masslist and Chemspider. Principal component analysis (PCA) was directly exported. Heatmap analysis of metabolites was performed using R (version 3.6.3) software.

**Volatile metabolites**

**Samples processing**

Each sample were selected 100 mg and placed in a 2 mL microcentrifuge tube. Two 3 mm steel beads were add in the tub. Then each sample was snap-freezed in liquid nitrogen. The freeze dried sample was ground using a Retsch mill (2 min, 25 Hz). Removing the steel beads, 1 mL dichloromethane were added and vortexed thoroughly, then centrifuged at 15000 rpm under room temperature for 10 min. The supernatant (1 µL) was analyzed by GC-MS.

**GC-MS analysis**

The samples were injected into a GC that is equipped with a quadrupole MS (Waters). An Agilent DB-5 (30mm x 0.25mm x 0.25 μm film thickness) column was used with a 3 min solvent delay and a flow rate of 1.5 mL/min. Injected samples were subjected to the following temperature program: Initial hold at 50°C for 2 min; a 8°C/min ramp to 150°C and hold for 3 min; a 10°C/min ramp to 240°C and hold 20 min. The injector temperature was maintained at 250°C. The inlet was operated in pulsed spitless injection mode. The GC–MS electron impact source was operated in scan mode with the MS source temperature at 230°C and MS Quad at 150°C.

**Supplementary Figures**


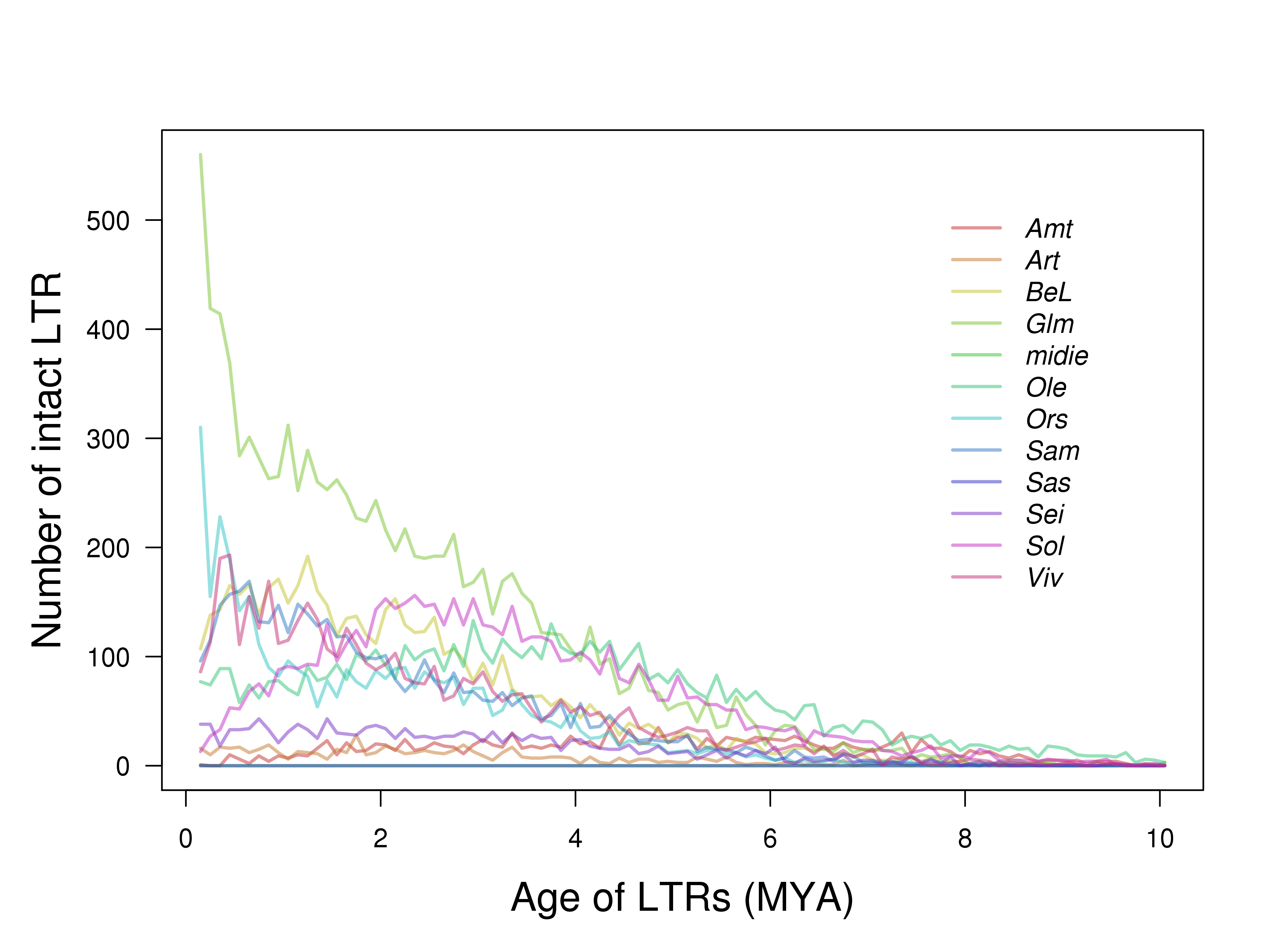


**Figure S1.** LTR insertion time of rosemary.

**
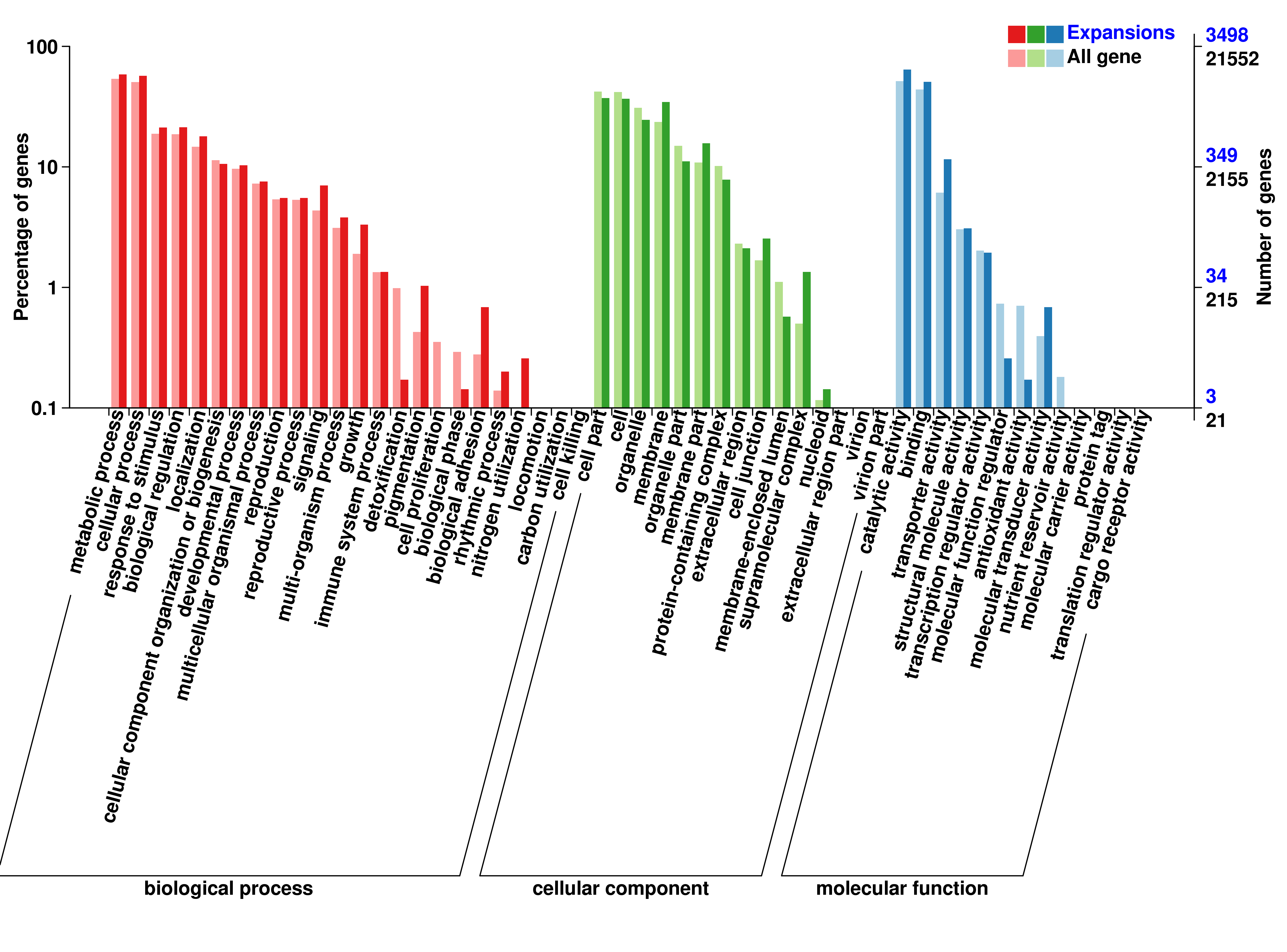
**

**Figure S2.** GO annotation counts of the expanded genes in rosemary.

**
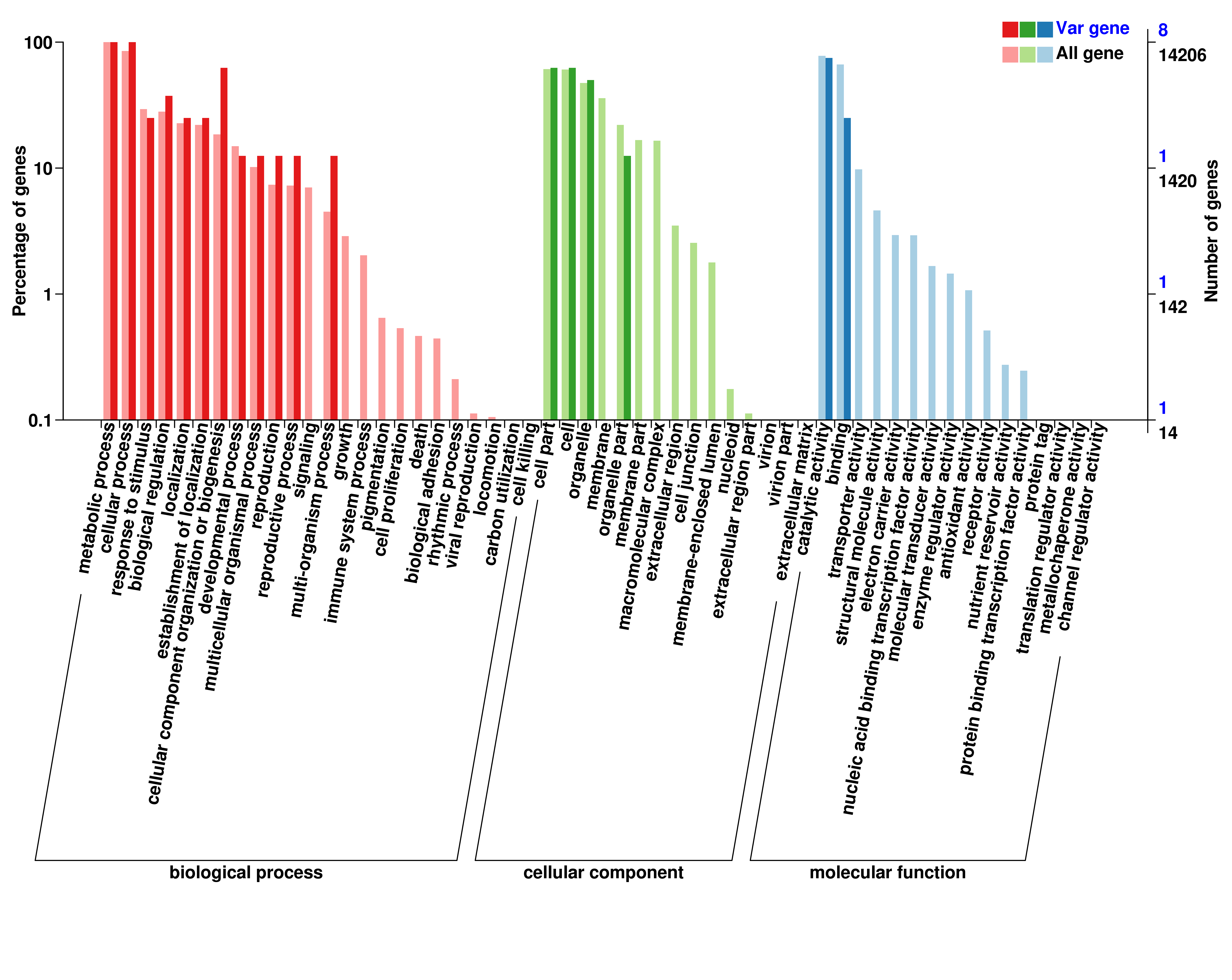
**

**Figure S3.** GO annotation counts for positive selective genes in rosemary.

**
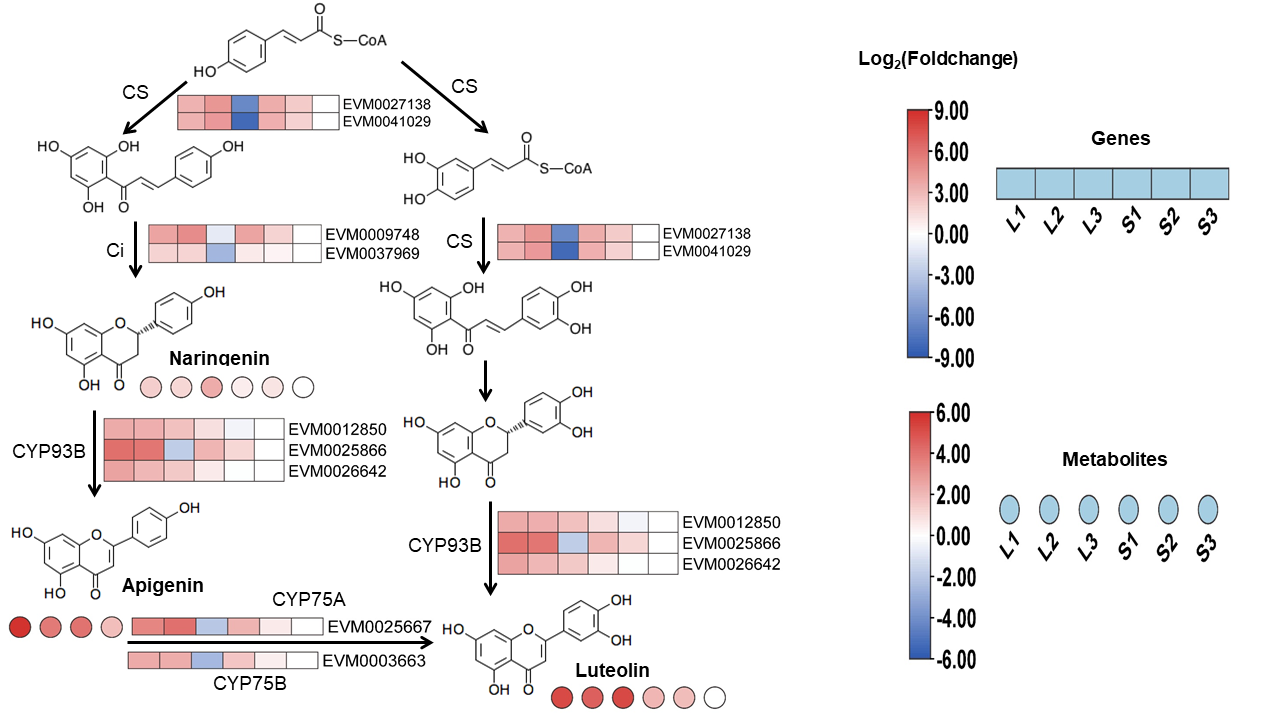
**

**Figure S4.** The biosynthetic pathway of flavonoids (naringenin, apigenin and luteolin. CS, chalcone synthase; Ci, chalcone isomerase; CYP75A, flavonoid 3',5'-hydroxylase; CYP75B, flavonoid 3'-monooxygenase; CYP93B, flavone synthase II.


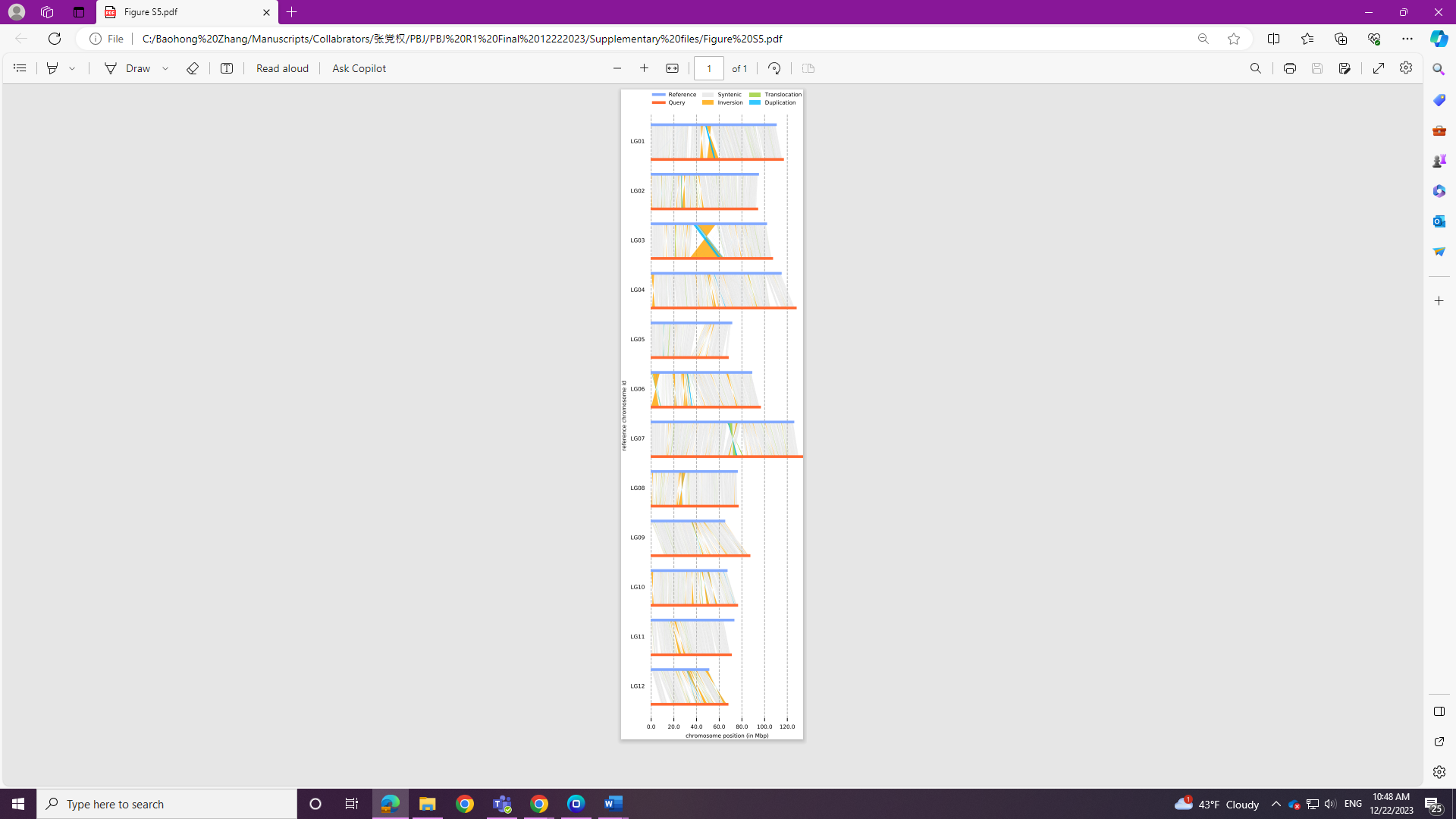


**Figure S5.** Synteny map and structural variation distribution between Albus-2 and Morocco genomes. Reference represents chromosome of Albus-2, and query represents chromosome of Morocco

**
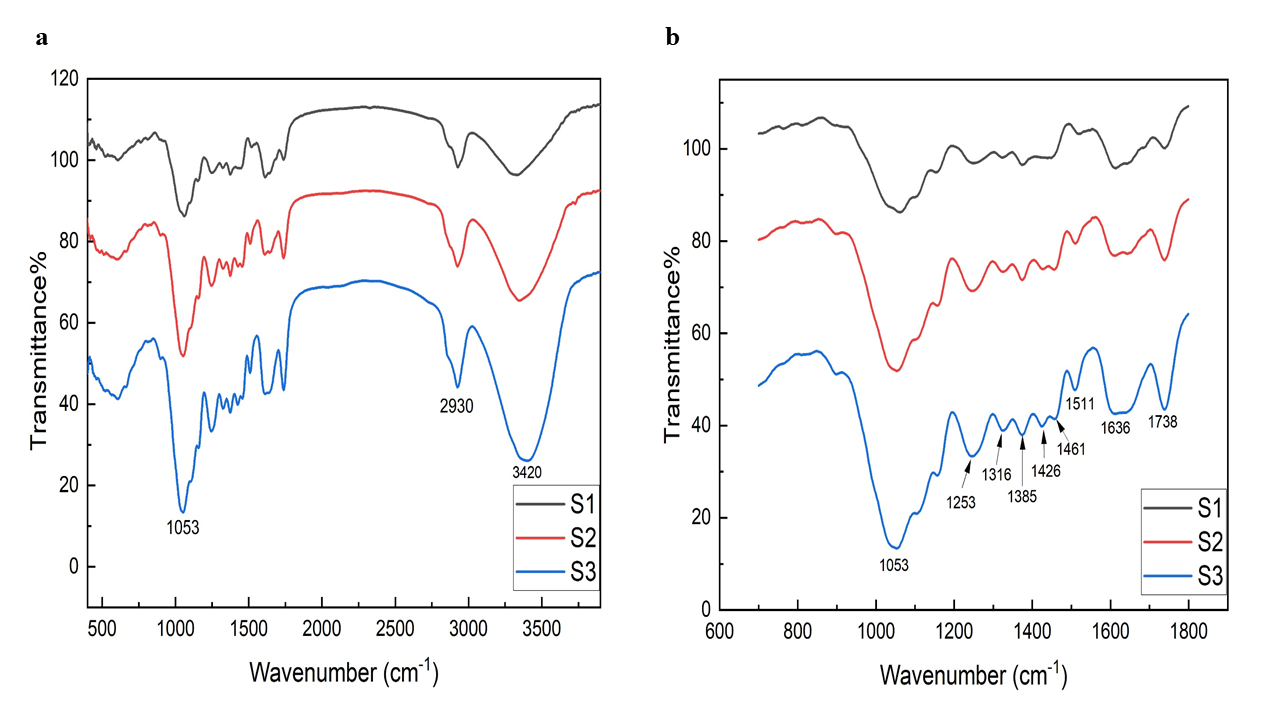
**

**Figure S6.** FTIR spectra of rosemary shoots under different lignification degrees. (**b**) The magnified view at the wavenumber 600 to 1800 cm^-1^. The peak at 1,053 cm^-1^ was assigned to polysaccharide of cell wall. The peak at 1,316 cm^-1^ was assigned to cellulose. The peaks at 1,385 and 1461 cm^-1^ were assigned to lignin, proteins and lipids.


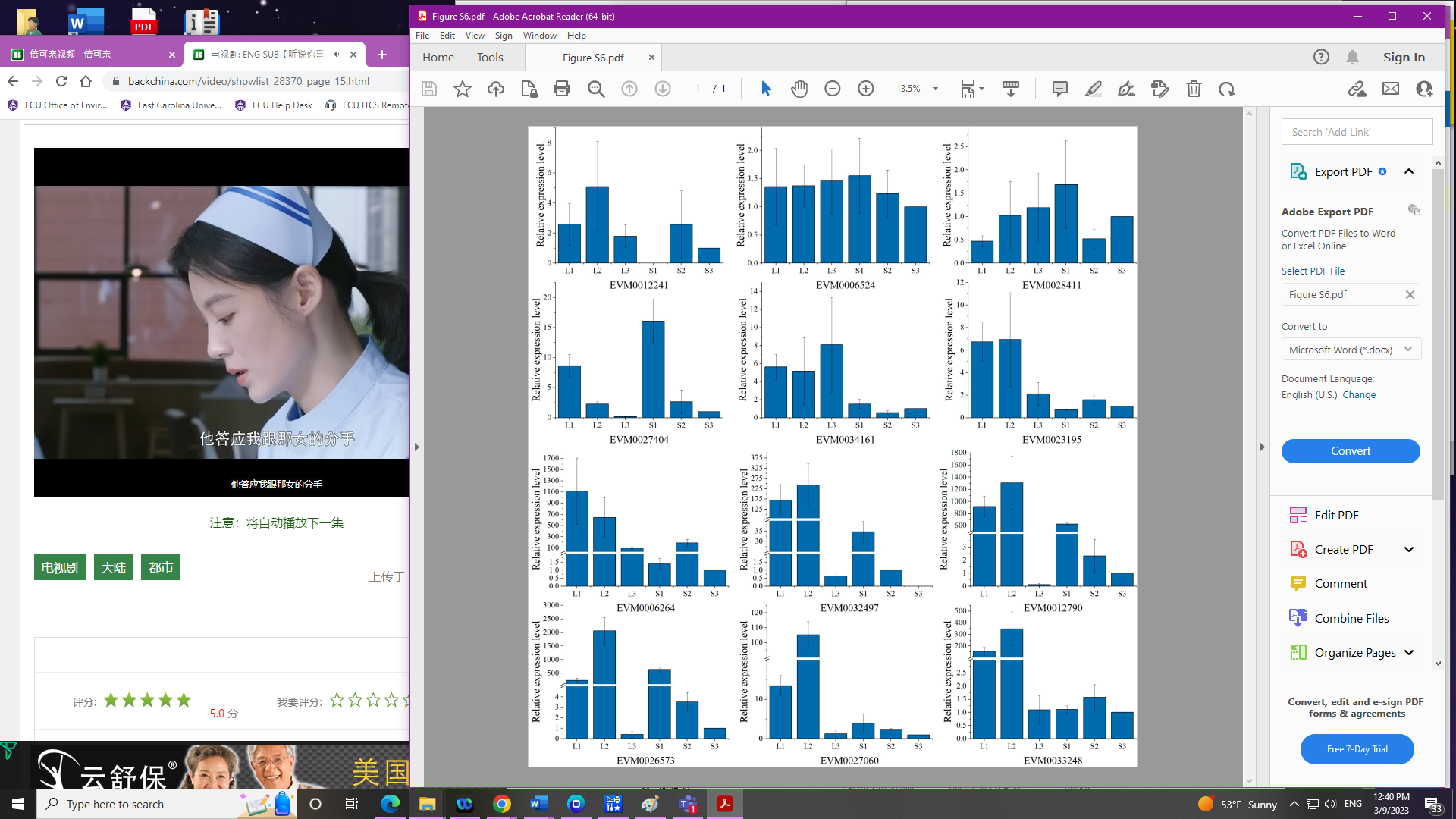


**Figure S7.** The relative expression level of selected genes determined by qRT-PCR analysis.

**Supplementary Tables**

**Table S1**. Statistics of the subread length distribution

| Length (bp) | Number | Total length (bp) | Average length (bp) |
| --- | --- | --- | --- |
| 2000~5000 | 271,670 | 931,207,330 | 3,428 |
| 5000~10000 | 329,575 | 2,436,347,668 | 7,392 |
| 10000~20000 | 724,108 | 11,348,205,101 | 15,672 |
| 20000~30000 | 1,116,285 | 27,848,105,937 | 24,947 |
| 30000~40000 | 898,269 | 31,191,372,422 | 34,724 |
| 40000~50000 | 583,384 | 25,977,144,126 | 44,528 |
| 50000~60000 | 279,177 | 15,138,446,167 | 54,225 |
| 60000~70000 | 92,294 | 5,902,688,879 | 63,955 |
| 70000~80000 | 23,760 | 1,755,631,700 | 73,890 |
| >=80000 | 9,979 | 909,642,980 | 91,156 |
| Total | 4,328,501 | 123,438,792,310 |  |

**Table S2**. Statistical information for gene family

| Species | Total gene number | Clusterer gene number | Total gene family | Unique gene family |
| --- | --- | --- | --- | --- |
| *O.europaea* | 39797 | 33539 | 13952 | 704 |
| *S.indicum* | 27148 | 23408 | 13175 | 385 |
| *S.lycopersicum* | 25571 | 23244 | 13246 | 358 |
| *O.sativa* | 38852 | 25781 | 12555 | 1933 |
| *A.trichopoda* | 16986 | 15187 | 11228 | 241 |
| *G.max* | 56044 | 46520 | 14870 | 1812 |
| *S.splendens* | 54008 | 45613 | 15696 | 1158 |
| *A.thaliana* | 27369 | 23284 | 12685 | 771 |
| *B.vulgaris* | 36014 | 22958 | 13027 | 1059 |
| *V.vinifera* | 26346 | 19390 | 12580 | 652 |
| *R.officinalis* | 46121 | 37464 | 15968 | 583 |
| *S.miltiorrhiza* | 30478 | 24866 | 14030 | 621 |

**Table S3.** Statistical information for genetic variations between Albus-2 and Morocco.

| Variation | Number |
| --- | --- |
| Single nucleotide polymorphism | 9,342,300 |
| Insertion/Deletion | 6,726,703 |
| Translocation | 6,497 |
| Inversion | 207 |
| Duplication | 25,354 |
| Copy number variation | 4,068 |
| Presence/absence variation | 9,058 |
| Total | 16,114,187 |

**Table S4**. Primers for the selected genes

| Gene | Forward | Reverse |
| --- | --- | --- |
| EIF-4A | ACGAGATGGGAATAAAGGAGGAG | ATCCTCACCCACACTTTTGCCTC |
| EVM0012241(Shikimate O-hydroxycinnamoyltransferase) | CGGTGGATTATTCGCTGGGA | GATGAATGGCGAGGGGTTGA |
| EVM0006524(Shikimate O-hydroxycinnamoyltransferase) | GCATATCGAGTACCAGCCCC | TTCGTGTCTTGGTCTTCGGG |
| EVM0028411(Rosmarinic acid synthase) | CACGTACCCCCTCATGCTTT | AGGCGATCTCTGTGTGTTGG |
| EVM0027404(Rosmarinic acid synthase) | GGCAATAGCCCGAATGG | CGAAACAAATAACTCCTCCCT |
| EVM0034161(Rosmarinic acid synthase) | GGAAGAAACGCCGAGTGG | CCTCCACGAACAATACGCC |
| EVM0023195(Farnesyl diphosphate synthase) | AGGGAGACGTTTATGGGGGT | CCAACATACGCTCGACCCAT |
| EVM0006264(Farnesyl diphosphate synthase) | GACAACTCTCAAGCACGGGA | ATGACAATGCCCTCGCAGAA |
| EVM0032497(CYP76AK7) | ACGCAGATAAATGGCTATACAATCC | ATTCAGCCCCTCCTTCAAGTTCC |
| EVM0012790(CYP76Ak8) | TGGATAGCGAGATTGATTTTGGAG | TCTGGAGTTGTTTGGATTCTGCC |
| EVM0026573(Pinene synthase) | GAGGAGGCCGGACATGAATC | CCAACGCCCCAAAAGTAGGA |
| EVM0027060(Pinene synthase) | GGACACTATTCGGAGCTGGG | CGCTTCAGCCAAATCTACCC |
| EVM0033248(Pinene synthase) | ATGTTGGGACAGCACCGTTT | GCGACCAATTCATCCGCAAA |
